# Beyond *p*‐Hexaphenylenes: Synthesis of Unsubstituted *p*‐Nonaphenylene by a Precursor Protocol

**DOI:** 10.1002/chem.202001531

**Published:** 2020-12-01

**Authors:** Ali Abdulkarim, Marvin Nathusius, Rainer Bäuerle, Karl‐Philipp Strunk, Sebastian Beck, Hans Joachim Räder, Annemarie Pucci, Christian Melzer, Daniel Jänsch, Jan Freudenberg, Uwe H. F. Bunz, Klaus Müllen

**Affiliations:** ^1^ Organisch-Chemisches Institut Ruprecht-Karls-Universität Heidelberg Im Neuenheimer Feld 270 69120 Heidelberg Germany; ^2^ InnovationLab Speyerer Str. 4 69115 Heidelberg Germany; ^3^ Kirchhoff-Institut für Physik Ruprecht-Karls-Universität Heidelberg Im Neuenheimer Feld 227 69120 Heidelberg Germany; ^4^ Centre for Advanced Materials Ruprecht-Karls-Universität Heidelberg Im Neuenheimer Feld 225 69120 Heidelberg Germany; ^5^ Max Planck Institute for Polymer Research Ackermannweg 10 55128 Mainz Germany

**Keywords:** conjugation, oligo-*para*-phenylene, pi interaction, precursor route

## Abstract

The synthesis of unsubstituted oligo‐*para*‐phenylenes (**OPP**) exceeding *para*‐hexaphenylene—in the literature often referred to as *p*‐sexiphenyl—has long remained elusive due to their insolubility. We report the first preparation of unsubstituted *para*‐nonaphenylenes (**9PP**s) by extending our precursor route to poly‐*para*‐phenylenes (**PPP**) to a discrete oligomer. Two geometric isomers of methoxylated *syn*‐ and *anti*‐cyclohexadienylenes were synthesized, from which **9PP** was obtained via thermal aromatization in thin films. **9PP** was characterized via optical, infrared and solid‐state ^13^C NMR spectroscopy as well as atomic force microscopy and mass spectrometry, and compared to polymeric analogues. Due to the lack of substitution, *para*‐nonaphenylene, irrespective of the precursor isomer employed, displays pronounced aggregation in the solid state. Intermolecular excitonic coupling leads to formation of H‐type aggregates, red‐shifting emission of the films to greenish. **9PP** allows to study the structure–property relationship of *para*‐phenylene oligomers and polymers, especially since the optical properties of **PPP** depend on the molecular shape of the precursor.

## Graphical abstract

**Unsubstituted** ***para*** **‐nonaphenylenes** were prepared via thermal aromatization of cyclohexadienylene precursors in thin films. The greenish fluorescence of the material is caused by pronounced aggregation in the solid state, in which electronic couplings leads to formation of H‐type aggregates. The emission bands of this small‐molecule are similar with respect to its polymer analogue.

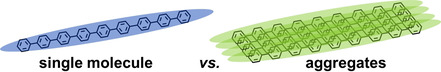

## Introduction

The structural motif of *para*‐phenylenes has experienced renewed interest in the last two decades, scientifically spurred by the synthesis of cyclo‐*para*‐phenylenes (CPPs), as well as the development of precursor routes yielding pristine, defect‐free poly‐*para*‐phenylene.[^1^, ^2^, ^3^] The lasting enthusiasm for materials with *para*‐phenylene moieties recently led to the development of curved and topologically new *para*‐phenylene derivatives, for example, catenated CPPs,[^4^, ^5^] CPP knots,[^4^, ^5^] CPP‐PPP hybrids,^[6]^ [2.2]paracyclophane‐containing cyclic OPPs,^[7]^ or spirobifluorene‐OPP ladder‐type hybrids.^[8]^ However, whereas several [*n*]CPPs of different diameters (with ring sizes *n*=5 to 18) have been synthesized,^[9]^ only little is known about their purely unsubstituted open‐chain congeners, the discrete oligo‐*para*‐phenylenes (OPP), exceeding hexaphenylenes in size.^[10]^

**Scheme 1:**
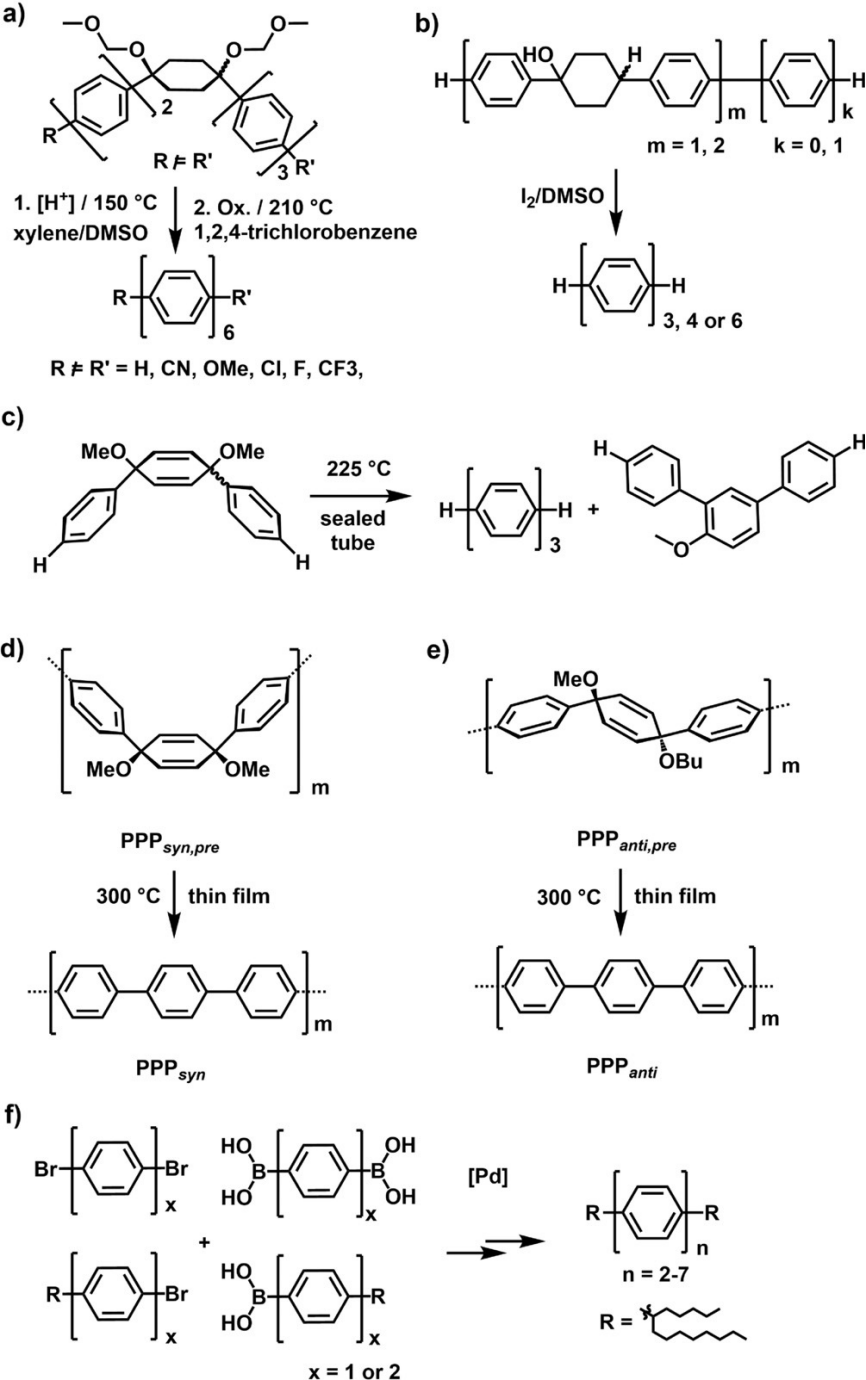
Previous synthesis of OPPs via precursor approaches (a–c), synthesis of **PPP** via *syn*‐ and *anti*‐configurated precursor polymers (d,e) and synthesis of terminally alkylated OPPs by Rathore et al. (f).

In contrast to curved π‐systems such as [*n*]CPPs, which exhibit a diminished tendency to aggregate and thus are accessible via solution chemistry, the synthesis of totally unsubstituted and “flat” OPPs (*n*>6) remains challenging due to their extremely low solubility^[11]^ impeding both formation of the phenylene chain as well as purification. For polymeric *para*‐phenylenes, this issue has been resolved through the development of precursor routes: non‐aromatic and non‐planar precursor polymers containing „masked“ phenylenes (e.g. substituted cyclohexadienylenes and cyclohexenylenes) are, as a key step, aromatized, overcoming the problem of solubility and yielding high‐molecular PPP. The quality of the products has appeared to depend sensitively upon on the nature of the precursor and the aromatization conditions. Similar to polymers, precursor strategies towards OPPs can be divided into two categories (Scheme 1): Methods of solution‐mediated aromatization (Scheme 1 a and b) suffer from necessary follow‐up steps to isolate and characterize the insoluble products as well as diminished yields;[^12^, ^13^] precursor routes to OPPs involving solely thermal solid‐state aromatization processes are challenging for small‐molecules. The problems during the thermal process are evaporation or melting resulting in undesired side‐reactions.^[14]^ For example, it was demonstrated that upon thermal demethoxylation of the methoxy‐masked *para*‐terphenyl at 225 °C in the bulk (cf. Scheme 1 c), a yield of only 70 % *para*‐terphenyl was obtained. The *meta*‐terphenyl was formed in 30 % yield due to a 1,2 phenyl shift.^[14]^ The easily evaporable methoxy‐masked *para*‐terphenyl makes the use of a sealing‐tube indispensable, in which the leaving‐groups (formally methoxy radicals) are trapped in the vessel and might cause additional side‐reactions. In contrast, no rearrangements were observed for our precursor polymer thin films (Scheme 1 d and e), in which dialkoxylated cyclohexadienylene moieties were thermally aromatized under nitrogen atmosphere and ambient pressure.[^1^, ^2^, ^3^] We concluded that aromatization was far more selective in thin films: the precursor polymers **PPP**_***syn,pre***_ and **PPP**_***anti,pre***_ did not melt in contrast to methoxy‐masked *para*‐terphenyl (cf. Scheme 1 c, *T*_m_=131 °C). To apply our protocol to discrete oligo‐*para*‐phenylenes, it is thus necessary to employ non‐volatile precursor oligomers that aromatize without dewetting or melting, furnishing pristine OPP films.

## Results and Discussion

We report the first synthesis of unsubstituted *para*‐nonaphenylene (**9PP**) via a precursor route. **9PP** can be considered as a model compound to study the underlying structure–property relationship of *para*‐phenylene oligomers and polymers, especially since the optical properties of **PPP** crucially depend on the precursor geometry[^1^, ^2^, ^3^] and the aromatization temperature.

**Scheme 2:**
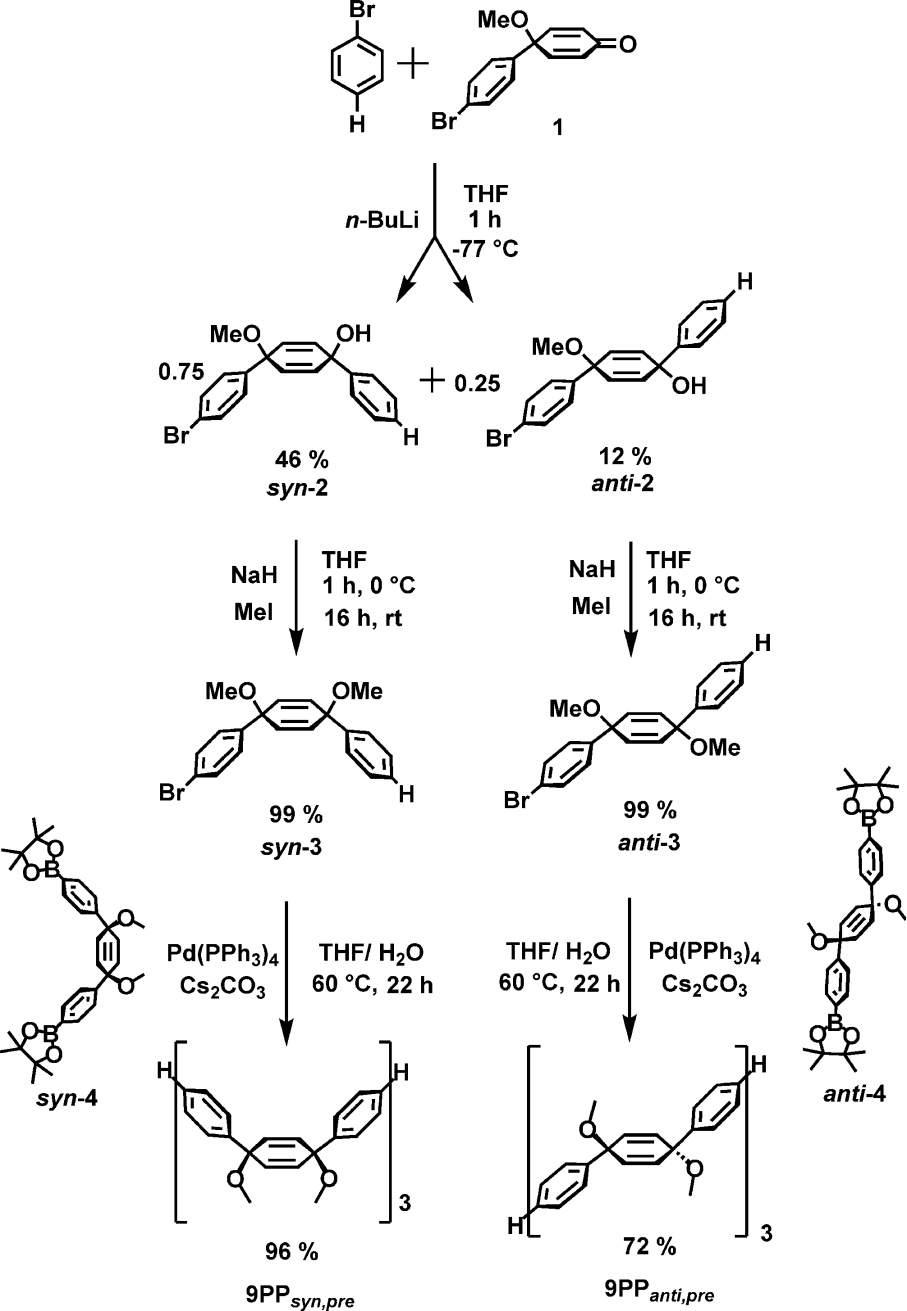
Synthesis of precursor oligomers **9PP**_***anti***,***pre***_ and **9PP**_***syn***,***pre***_.

The synthesis of **9PP** started with the addition of phenyl lithium to ketone^[15]^ **1** to yield a mixture of the *syn*‐ and *anti*‐isomers ***syn***‐/***anti***‐**2** in a ratio of 3:1 (Scheme 2). After separation by column chromatography on silica, 46 % of ***syn*** **‐2** and 12 % of ***anti‐2*** were successfully isolated. Subsequently, nearly quantitative alkylation employing sodium hydride and methyl iodide furnished methoxylated ***syn‐3*** (99 %, colorless oil) and ***anti‐3*** (99 %, colorless crystals, for crystal structure see Figure S20, Supporting Information). Both isomers were subjected to Suzuki coupling reactions utilizing previously prepared diborylated masked terphenyls ***syn‐4*** and ***anti‐4***[^1^, ^2^, ^3^] yielding precursor oligomers **9PP**_***anti,pre***_ (72 %) and **9PP**_***syn,pre***_ (96 %). Work‐up included precipitation in methanol and diethyl ether as well as chromatography over a short aluminum oxide column. The lower yield of the *anti*‐isomer **9PP**_***anti,pre***_ can be ascribed to its lower stability in solution compared to the corresponding *syn*‐isomer. Due to their non‐planar geometries, both precursors are soluble in chloroform or dichloromethane enabling solution processing.

**Scheme 3:**
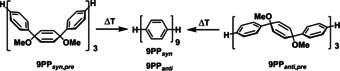
Thermal aromatization furnishing **9PP_syn_** and **9PP_anti_** starting from the corresponding oligophenylene precursor isomers.

The demethoxylation of the precursor oligomers **9PP**_***anti,pre***_ and **9PP**_***syn,pre***_ (Scheme 3) was studied via TGA/DSC analyses in the bulk (Figure S1, Supporting Information). The respective temperatures *T*_arom_ amounted to 260 °C for **9PP**_***syn,pre***_ and 250 °C **9PP**_***anti,pre***_, as displayed by sharp exothermic peaks in the DSC profiles accompanied by a mass loss in the thermograms. *T*_arom_ of the two isomers only differed by 10 °C (250 °C vs. 260 °C), while those of their polymeric counterparts (**PPP**_***syn***_ vs. **PPP**_***anti***_) deviated more from each other (Δ*T*_arom_=55 °C; **PPP**_***anti***_ =222 °C vs. **PPP**_***syn***_ =277 °C).[^1^, ^2^, ^3^] Both compounds **9PP**_***anti,pre***_ and **9PP**_***syn,pre***_ did neither sublime nor melt in the relevant temperature regime up to 300 °C and thus fulfilled the above‐mentioned criteria for solid‐state aromatization. Smaller methoxy‐masked OPPs prepared in our group, such as cyclohexadienylene‐based heptaphenylene precursors, or different geometric isomers of nonaphenylene precursors (see Figure S8 for structures, Supporting Information), had too low melting points or sublimed during annealing.

**Figure 1:**
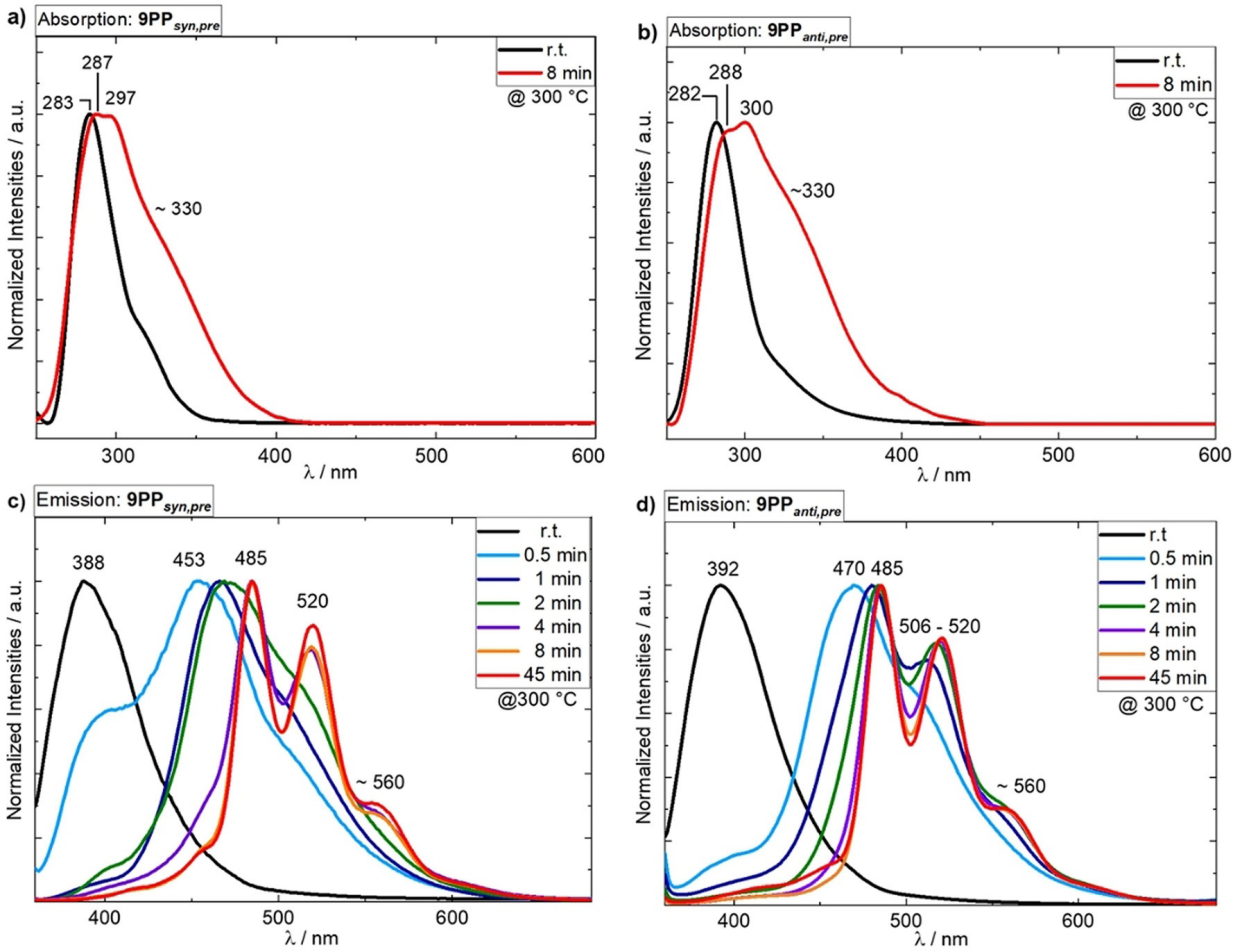
Absorption spectra of **9PP**_***syn***,***pre***_ (a) and **9PP**_***anti***,***pre***_ (b) in thin films measured before and after treatment at 300 °C for eight minutes. Time‐dependent evolution of emission spectra (λ_exc_=300 nm) of **9PP**_***syn***,***pre***_ (c) and **9PP**_***anti***,***pre***_ (d) while annealing at 300 °C.

For an analysis of the aromatization process (Scheme 3), time‐dependent absorption and emission spectroscopy as well as Fourier transform infrared (IR) spectroscopy of the spin coated thin films was conducted.[^1^, ^2^, ^3^] Thin films (<7 nm) of each precursor were deposited on quartz glass via spin coating. Both films showed an absorption maximum at 282/283 nm. Absorption spectra were taken after heating the samples to 300 °C for different time intervals (1, 2, 4, 8 and 45 minutes; see Figure 1 for initial spectra and absorption after 8 min; see Figures S5 and S6, Supporting Information, for time‐dependent evolution of the spectra).^[16]^

The formation of **9PP**_***syn/anti***_ resulted in a gradual bathochromic shift of the absorption maximum from 283 nm to 287 and 297 nm for the *syn*‐isomer and from 282 nm to 288 and 300 nm for the *anti*‐isomer, respectively, as well as formation of a shoulder at about 330 nm for both derivatives after 8 min of thermal treatment (Figure 1 a and b).

These solid‐state absorption maxima were at much lower wavelengths than expected when compared to literature known OPPs in solution.[^13^, ^17^, ^18^, ^19^, ^20^, ^21^, ^22^, ^23^, ^24^, ^25^, ^26^]

Previous studies of *para*‐alkylated and thus soluble OPPs by Rathore et al. (see Scheme 1 f) showed a gradual redshift with increasing number of phenylene units (n).^[17]^ The linear dependency of 1/*n* with 1/*λ* for the series of biphenyl up to *p*‐heptaphenylene (*λ*_abs_=326 nm) allows for an extrapolation of the optical properties to hitherto unknown higher oligomers and polymers with the theoretical value of ca. *λ*_abs, theor._=334 nm and *λ*_em, theor._=417 nm for **9PP**.^[17]^ While the linear regression holds for optical properties in dilute solutions, in which only in‐chain conjugation is considered, one cannot neglect the intermolecular packing within the condensed phase.

Based on Kasha's fundamental work,^[27]^ Spano et al. introduced the classification of H‐ and J‐type coupling,[^28^, ^29^, ^30^, ^31^, ^32^] which was later extended by other groups.[^33^, ^34^] As such, J‐type coupling describes the intramolecular interactions of phenylenes, a consequence of their torsion, as well as an intermolecular interaction in a head‐to‐tail fashion of the chromophores. H‐type coupling represents the inter‐chain interactions of chromophores oriented side‐by‐side, mostly dominated by their inter‐chain distances and respective orientation to each other.[^28^, ^29^, ^30^, ^31^, ^32^, ^35^, ^36^, ^37^] More pronounced J‐type coupling leads to a bathochromic shift of the absorption, whereas increasing H‐type coupling results in a hypsochromic shift. Regarding emission spectra, both H‐ and J‐type couplings are additive and lead to a bathochromic shift.[^29^, ^30^, ^31^, ^32^, ^35^, ^38^, ^39^, ^40^, ^41^, ^42^, ^43^, ^44^, ^45^] Furthermore, distinction of face‐to‐face vs. edge‐to‐face orientations of the chromophores is necessary, since they result in different emission characteristics. While edge‐to‐face orientations lead to fine‐structured emission bands (H‐type coupling), face‐to‐face coupling results in unstructured, even more redshifted emission bands (excimer‐like coupling).^[34]^

For crystalline rod‐like, unsubstituted (without solubilizing side‐chains) hydrocarbon oligomers, J‐type‐aggregation plays a minor role, since they are usually arranged side‐by‐side,[^34^, ^46^, ^47^] to allow for dense packing of the oligomer backbones, balancing their quadrupole moment most effectively.^[48]^ Rod‐shaped oligomers such as *para*‐phenylenes (*n*=2–6), *para*‐phenylene vinylenes, acenes and oligothiophenes pack in an edge‐to‐face arrangement. Mostly independent of their processing technique, these oligomers arrange in a herringbone fashion to balance the π–π‐overlap with enthalpic gain by dense packing.[^34^, ^49^, ^50^]

Analyzing our annealing process in more detail, we observe that the absorption maximum (see Supporting Information Figure S5) redshifts from 283 nm (*syn*‐precursor isomer) to ca. 293 nm after only 1 min of thermal treatment. This is followed by a blueshift to 289 nm after an additional minute, without any further significant changes for the further annealing period (although additional bands appeared). We ascribe the first redshift mainly to the extension of the π‐systems due to aromatization/demethoxylation. The following blueshift of the absorption maximum is assigned to aggregation effects resulting from morphological changes in the thin films. Both processes—aromatization and aggregation—cannot be well separated. We observe a similar trend of absorption maximum shifts for the corresponding *anti*‐isomer. Starting from 282 nm, a successive redshift to 288 nm is observed within the first 8 min of the annealing process. In this case, aromatization and morphological rearrangement occur simultaneously. The opposite effects regarding the absorption maxima—redshift due to aromatization and blueshift related to aggregation—result in a smaller total redshift compared to **9PP**_***syn***_. Nevertheless, after 45 min, the absorption maximum of **9PP**_***anti***_ is blueshifted from 288 to 284 nm indicative of H‐type coupling. This is not observed for **9PP**_***syn***_ due to the observed dewetting *(*vide infra*)*. Compared to the calculated value of absorption for **9PP** in solution (334 nm), the main absorption bands are hypsochromically shifted. Thus, the major aggregation in **9PPs** comprises H‐type coupling, since the annealing process displays a distinct hypsochromic shift after the first aromatization‐caused redshift. Comparing the absorption maxima to the calculated values, a hypsochromic shift of all main bands is observed. The interpretation of solid‐state inter‐chain interactions of semi‐ or (poly)crystalline films is, however, severely hampered compared to single crystalline materials. Thus, we cannot exclude the presence of non‐ or less coupled **9PP** strands. This would result in optical responses similar to single‐chain‐like behavior in diluted solutions, which may be attributed to the absorption shoulders observed at ≈330 nm.

The annealing process was also monitored via fluorescence spectroscopy of thin films (Figure 1, bottom), allowing for the same conclusion. Both masked precursors emitted at around 390 nm (**9PP**_***syn,pre***_: 388 nm; **9PP**_***anti,pre***_ 392 nm). After heating the *syn*‐isomer for periods as short as 30 seconds, the emission band at ca. 390 nm was reduced to roughly half of its original intensity. For the *anti*‐isomer, this band disappeared almost completely, indicating faster initial aromatization. After 30 s of further heating, the initial emission band of both precursors disappeared completely reflecting complete precursor consumption. Moreover, both isomers of thermally generated **9PP** emit at longer wavelengths compared to the postulated emission maximum in solution, indicating strong aggregation. New bands at 453 and 470 nm appeared, which were redshifted with increasing annealing time. After 4 min the spectra for both isomers were superimposable with fine‐structured emission bands at 485, 520 and 560 nm and greenish emission. The emission maximum at 485 nm is redshifted by about 68 nm compared to the extrapolated value from solution‐based measurements (≈417 nm), although some maxima with low intensities are observable at around 430 and 450 nm. This might be due to imperfect crystallization processes.^[51]^ Lifetime measurements (Section S4, Supporting Information) support that these weak emission bands may stem from single‐strand emission rather than aggregate emission found for the three major emission maxima.[^34^, ^51^]

The overall structure of the emission bands (either structureless or well‐resolved) allows one to distinguish between classic H‐type coupling (excitonic coupling) and excimer‐like coupling (electronic coupling) (vide supra).^[34]^ The latter one is caused by co‐facial π–π‐overlaps leading to large bathochromic shifts and unstructured emission bands attributed to the partial intermolecular charge‐transfer character of the optical transition. This leads to different degrees of intermolecular separation in the ground and first excited state, hence the coupling of low‐frequency breathing modes to the electronic transition, which is different from the intramolecular vibronic coupling of (purely) H‐type aggregates in the herringbone arrangement. Thus, the absence of those charge‐transfer contributions in the transition leads to bathochromically shifted, but well‐resolved emission band shapes of a relaxed exciton.^[34]^

Taking into account that most rod‐shaped unsubstituted hydrocarbons arrange in a herringbone fashion and combining this with our observation of well‐resolved emission bands, we suppose a classical H‐type coupling rather than an excimer‐type coupling.^[33]^

Moreover, all emission bands of **9PP**_***syn/anti***_ were identical to those of thermally generated **PPP**_***anti***_ ^[2]^ and even more redshifted compared to those of **PPP**_***syn***._ ^[1]^ Thus, the expectations of OPPs as a blue‐emitting bulk material can be disproved. Since the same emission spectra were obtained, regardless of the geometry of the precursor, we conclude that the different optical properties of the polymers (see Supporting Information, Figure S7), which emit at 485, 520 and 560 nm for **PPP**_***anti***_ and 426, 450, 485 and 560 nm for **PPP**_***syn***_, respectively, are a consequence of their differing morphologies and solid‐state order. Furthermore, the estimated timescales of the aromatization process for both isomers are comparable to each other and with that of **PPP**_***anti***_ (5 min) and much faster than that of **PPP**_***syn***_ (40 min).[^2^, ^3^]

For a detailed analysis of the aromatization process, Fourier transform infrared (IR) spectroscopy was conducted. Since this technique has been employed to quantify the conversion process of insoluble materials like their polymeric analogues, ≈40 nm thick films of **9PP**_***syn,pre***_ and **9PP**_***anti,pre***_ were spin‐coated on SiO_2_/Si for comparison with our previous studies.[^2^, ^3^] Unfortunately, the *syn*‐isomer showed undesired dewetting below the aromatization temperature (*T*_dewet_<180 °C), which, apart from different substrates, surface energies and film thicknesses, ^[52–56]^ can be due to the different molecular shapes and stacking of the **9PP**_***syn,pre***_ rotamers (see Figure S2, Supporting Information). Since rotation about the single bonds of **9PP**_***anti,pre***_ yields rotamers of roughly the same molecular shape (all of the brick‐type), dewetting of this isomer up to 300 °C is most likely suppressed. Thus, only the IR spectra of **9PP**_***anti,pre***_ are discussed—the corresponding results of the *syn*‐isomer are depicted in the Supporting Information (Figure S14).

**Figure 2:**
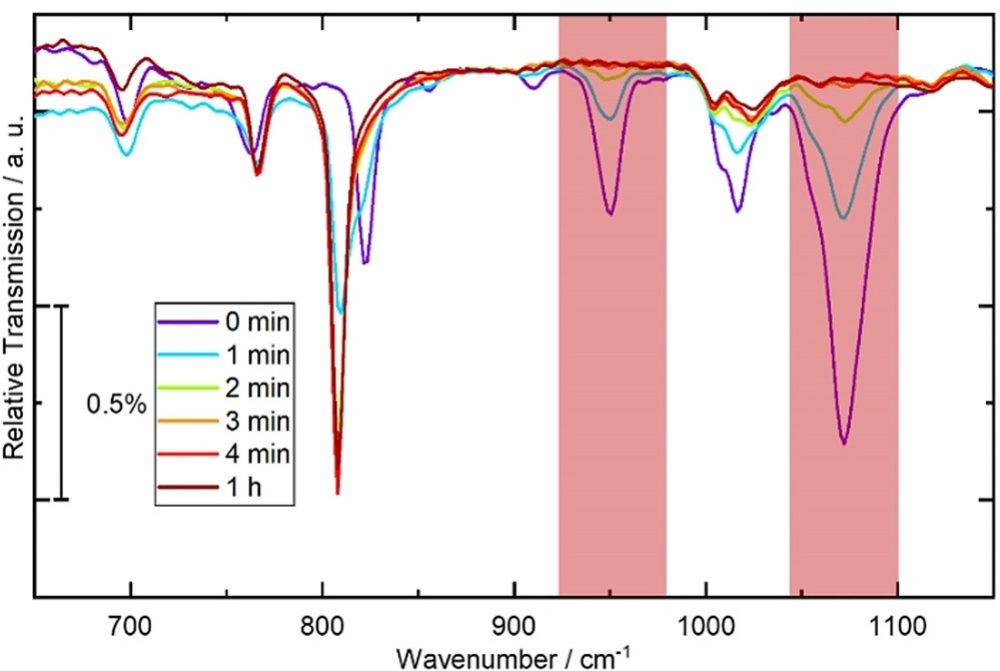
Time‐resolved infrared spectroscopy (finger‐print region) while annealing **9PP**_***anti***,***pre***_ at 300 °C. Red squares highlight the symmetric (950 cm^−1^) and asymmetric (1076 cm^−1^) C−O bond stretching vibrations, which gradually lose intensity.

Transmission IR spectra of **9PP**_***anti,pre***_, annealed stepwise at 250 °C and at 300 °C, illustrate the gradual transition from the precursor to **9PP**_***anti***_ (300 °C: Figure 2; 250 °C: see Figure S10, Supporting Information), with the product spectra being in good agreement with the DFT calculated ones (Supporting Information). The IR spectra confirmed that aromatization at 300 °C leads to quantitative conversion after only 3–4 min, which is in good agreement with the timescale deduced from the optical spectra. At 250 °C, 1 h of temperature treatment was necessary to achieve quantitative product formation (Figure S10, Supporting Information). The processes were monitoring by the two absorption bands at ca. 1076 and 950 cm^−1^, which were assigned to the symmetric and asymmetric stretching C−O bonds, only observable in the methoxylated precursor compounds.

Comparing the nonaphenylene precursor with the precursor polymer having *anti*‐configuration, time periods needed for full aromatization at 300 °C were similar (**PPP**_***anti***_: *t*_arom_=5 min).

Angle‐dependent IR spectra revealed a change of preferred molecular orientation: **9PP** was oriented edge‐on (for rod‐type molecules sometimes called “end‐on”)^[50]^ with respect to the substrates although its precursor showed a face‐on orientation before temperature treatment (for details see Figure S11, Supporting Information).

**Figure 3:**
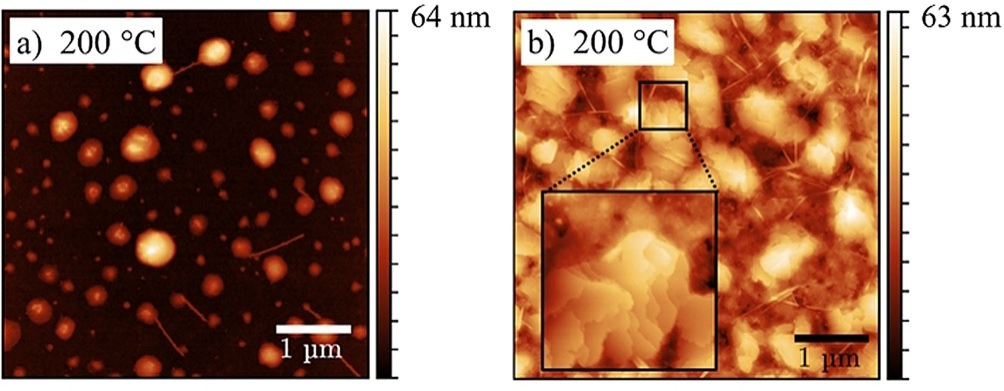
AFM‐images of a) **9PP**_***syn***_ (dewetting) and b) **9PP**_***anti***_ (crystalline), inset: 0.7 μm x 0.7 μm.

Besides IR spectroscopic analysis of **9PP** and its optical characterization, the formation of **9PP**_***anti***_ was observed via MALDI mass spectrometry of aromatized films coated with TCNQ (7,7,8,8‐tetracyanoquinodimethane) as a matrix (see Figure S16), providing further structure proof. Aromatization/demethoxylation is quantitative in the bulk as well as evidenced by solid state ^13^C NMR spectra of both precursor isomers and thermally generated **9PP**_***syn/anti***_ (Figures S29–33, Supporting Information). Atomic force micrographs of the aromatized **9PP**_***anti***_ on SiO_2_/Si (Figure 3) exhibited highly crystalline domains, comparable in size, shapes, mounds and terrace formation to highly ordered *para*‐hexaphenylene films.^[48]^ For *para*‐hexaphenylene in thin films, mound and terrace formation is a consequence of molecular edge‐on (“end‐on”) orientations with different tilt angles of molecules aligned in a herringbone motif.^[48]^ Thus, the observed morphology is in agreement with both the H‐type aggregation deduced from the optical properties as well as the preferential orientation deduced from IR spectroscopy.

## Conclusions

In summary, we successfully applied our previously developed precursor approach yielding poly‐*para*‐phenylenes to prepare small‐molecule oligophenylenes in thin films, as demonstrated by the synthesis of nonaphenylenes **9PP**_***syn/anti***_. The choice of the precursor is vital to obtain pristine and defect‐free materials. A too low melting point of the precursor results in undesired phenyl shifts upon thermal aromatization, while its molecular weight needs to be large enough to avoid sublimation and dewetting, upon applying the thermal stimulus. However, basically any larger monodisperse oligophenylene is now accessible by combining methoxylated *anti*‐cyclohexadienylenes as masked 1,4‐phenylene building blocks with the desired number of phenylene spacers and end‐cappers, bridging the gap between solution‐phase synthesis of the smaller oligophenylenes up to *p*‐hexaphenylene and the phenylene polymers. Although the notion of unsubstituted oligo‐ and poly‐phenylenes as blue emitters is evoked in many publications, it may only hold for hypothetical solutions, as the insoluble materials are prone to severe aggregation. The nonaphenylenes prepared by heating the thin films to 250 °C or 300 °C display significant H‐type aggregation utilizing Spano's classification. This due to the π‐stacking of the aromatic systems in the ordered thin films as evidenced by angle‐dependent IR spectrometry as well as atomic force microscopy, resulting in greenish emission. A key toward blue emitting oligophenylenes would thus be the suppression of intermolecular interactions. This might be achievable by dilution in a (polymeric) host not prone to degradation at 300 °C or, alternatively, embedding oligophenylene segments into a rigid organic framework. These approaches will be reported in due course.

## Experimental Section

All commercially available chemicals were used without further purification unless otherwise noted. The reactions were performed using standard vacuum‐line and Schlenk techniques. Workup and purification of all compounds were performed under air and with reagent‐grade solvents.

**Synthesis of 1**: To a solution of the 4‐bromo‐4’‐hydroxybiphenyl (12.5 g, 50.2 mmol, 1.00 equiv.) in MeOH (1.00 L) was added (diacetoxyiodo)benzene (20.0 g, 62.7 mmol, 1.25 equiv.) at room temperature under argon. After stirring for 72 h, the mixture was filtered, and the filtrate was concentrated in vacuo. The obtained residue was washed with cold methanol. The residue was purified by silica‐gel column chromatography (*n*‐hexane/EtOAc 10:1, v/v) to give the title compound (12.1 g, 43.2 mmol, 86 %). Alternatively, the residue was crystalized from diethyl ether on large scale (ca. 12 g). ^1^H NMR (300 MHz, CDCl_3_) δ [ppm]=7.38 (dd, *J=*8.7 Hz, 4 H), 6.55 (dd, *J=*10.2 Hz, 4 H), 3.40 (s, 3 H). ^13^C NMR (75 MHz, CDCl_3_) δ [ppm]=185.3, 150.0, 137.5, 132.0, 130.4, 127.6, 122.5, 76.3, 53.0. Analytical data in accordance with literature.^[15]^

**Synthesis of** ***syn*** **‐ and** ***anti*** **‐3**: Bromobenzene (12.4 g, 8.25 mL, 78.8 mmol, 2.20 equiv.) was dissolved in THF (200 mL) and the solution cooled to −77 °C. *n‐*BuLi (2.5 m in hexanes, 31.5 mL, 78.8 mmol, 2.20 equiv.) was added dropwise over 10 min. After stirring the reaction mixture for another 30 min, 4‐(4‐bromophenyl)‐4‐methoxycyclohexadien‐1‐one (**1**) (10.0 g, 35.8 mmol, 1.00 equiv.), dissolved in THF (50 mL), was added dropwise via syringe at −77 °C. The resulting reaction mixture was stirred for 1 h at −77 °C. The reaction was quenched by addition of water (50 mL) and the reaction mixture was allowed to warm to room temperature. EtOAc (70 mL) was added and the aqueous phase was extracted with EtOAc (3x 40 mL). The combined organic phases were washed with brine (100 mL), dried over sodium sulfate, and the solvent was removed under reduced pressure. The isomers were separated by a short column chromatography (SiO_2_, CH_2_Cl_2_/petroleum ether 50:50, v/v) yielding the *anti*‐product (*anti*‐**2**) as colorless solid (1.5 g, 4.3 mmol, 12 %) and the *syn*‐isomer (*syn*‐**2**) as colorless oil (5,9 g, 22.8 mmol, 46 %). Due to their intrinsic instability with respect to acidic conditions (silica), the column chromatography need to be done quickly. Both isomers were slightly contaminated with further by‐products, which was why methylation was carried out without further purification.

NaH (690 mg, 60 % in mineral oil, 17.2 mmol, 4.00 equiv.) was suspended in 100 mL dry THF and cooled to 0 °C. *anti*‐**2** (1.54 g, 4.31 mmol, 1.00 equiv.) was added to the cooled suspension. After 1 h of stirring, methyl iodide (1.07 mL, 17.2 mmol, 4.00 equiv.) was added. The mixture was warmed to room temperature and stirred for 16 h. The reaction was quenched with water and diluted with diethyl ether (50 mL). After layer separation, the aqueous layer was extracted with diethyl ether three times. The combined organic layers were washed with brine, dried over magnesium sulfate and the organic solvents were removed under reduced pressure. The crude product was purified by column chromatography (SiO_2_, EtOAc/petroleum ether 10:90, v/v) yielding the *anti*‐product (*anti*‐**3**) as colorless solid (1.58 g, 4.27 mmol, 99 %). Mp: 127 °C, ^1^H NMR (300 MHz, CD_2_Cl_2_) δ [ppm]=7.63–7.23 (m, H_arom_., 9 H), 6.18–6.00 (m, H_olefin.,_ 4 H), 3.27 (m, ‐OMe, 6 H). ^13^C NMR (75 MHz, CD_2_Cl_2_) δ [ppm]=143.6, 143.1, 134.0, 133.6, 131.4, 128.4, 128.3, 128.1, 127.6, 127.5, 126.2, 121.4, 74.7, 74.6, 51.9. FTIR, $\tilde{\nu }$ [cm^−1^]: 3088, 3071, 3059, 3037, 3028, 2983, 2972, 2945, 2936, 2903, 2893, 2824, 1601, 1588, 1479, 1467, 1446, 1402, 1281, 1261, 1224, 1176, 1068, 1054, 1018, 1007, 948, 910, 828, 818, 792, 771, 759, 716, 696, 674, 629, 566, 558, 542. Elemental analysis calcd. for C_20_H_19_BrO_2_: C=64.70; H=5.16; 2; found: C=65.55; H=5.20. Crystals were obtained by diffusion method using diethyl ether and petroleum ether. Crystal data: section 3 in Supporting Information.

NaH (1.61 g, 60 % mineral oil, 66.9 mmol, 4.00 equiv.) was suspended in 200 mL dry THF and cooled to 0 °C. *syn*‐**2** (5.89 g, 16.5 mmol, 1.00 equiv.) was added to the cooled suspension. After 1 h of stirring, methyl iodide (4.17 mL, 66.9 mmol, 4.00 equiv.) was added. The mixture was warmed to room temperature and stirred for 16 h. The reaction was quenched with water and diluted with diethyl ether (50 mL). After layer separation, the aqueous layer was extracted with diethyl ether three times. The combined organic layers were washed with brine, dried over magnesium sulfate and the organic solvents were removed under reduced pressure. The crude product was purified by column chromatography (SiO_2_, CH_2_Cl_2_/petroleum ether 30:70, v/v) yielding the *syn*‐product as colorless oil (*syn*‐**3**) (6.15 g, 15.6 mmol, 99 %). ^1^H NMR (300 MHz, CDCl_3_) δ [ppm]=7.52–7.27 (m, H_arom_, 9 H), 6.22–6.05 (m, H_olefin._, 4 H), 3.47 (m, ‐OMe, 6 H), ^13^C‐NMR (75 MHz, d_2_‐DCM) δ [ppm]=143.6, 134.1, 134.3, 133.4, 131.3, 128.3, 128.0, 127.5, 126.1, 121.3, 74.3, 74.2, 51.8, 51.7. FTIR $\tilde{\nu }$ [cm^−1^]: 3086, 3063, 3051, 3031, 2984, 2966, 2936, 2929, 2896, 2823, 2812, 1600, 1586, 1481, 1448, 1398, 1258, 1228, 1184, 1173, 1070, 1024, 1010. 948, 822, 756, 751, 697, 662, 613, 522. Elemental analysis calcd. for C_20_H_19_BrO_2_: C=64.70; H=5.16; found: C=66.33; H=5.40.

**Synthesis of 9PP**_***syn,pre***_: Monobromide *syn* **‐3** (205 mg, 551 μmol, 3.00 equiv.), diboronate ***syn*** **‐4** ^[1]^ (100 mg, 184 μmol, 1.00 equiv.), cesium carbonate (299 mg, 919 μmol, 5.00 equiv.), and tetrakis(triphenylphosphine)palladium (21.2 mg, 18.4 μmol, 10 mol‐%) were dissolved in 11.0 mL of degassed 10:1 THF/H_2_O. The solution was heated to 60 °C for 22 h. After cooling to rt, the aqueous layer was separated and the organic layer was precipitated into methanol and the product was collected via filtration. The crude product was further filtered through a short plug of aluminium oxide using EtOAc/petroleum ether (50:50, v/v) yielding the trimer (**9PP**_***syn,pre***_) as an off‐white solid (115 mg, 132 μmol, 94 %). ESI‐MS(+) (*m*/*z)*: calcd. for C_60_H_56_Br_2_O_2_ [Na+] 895.40, found 895.40. Elemental analysis calcd. for C_60_H_56_Br_2_O_2_: C=82.54; H=6.47; found: C=81.98; H=6.55, ^1^H NMR (300 MHz, CD_2_Cl_2_) δ [ppm]=7.68–7.24 (m, H_arom_., 26 H), 6.18–6.10 (m, H_olefin.,_ 12 H), 3.50–3.39 (m, ‐OMe, 18 H),_._^13^C NMR: due to solubility issue not recordable. FTIR $\tilde{\nu }$ [cm^−1^]: 3083, 3058, 3026, 2981, 2977, 2935, 2897, 2820, 1601, 1494, 1081, 949, 823, 756, 699, 662.

**Synthesis of 9PP**_***anti,pre***_: Monobromide *anti* **‐3** (205 mg, 551 μmol, 3.00 equiv.), diboronate ***anti*** **‐4** ^[2]^ (100 mg, 184 μmol, 1.00 equiv.), cesium carbonate (299 mg, 919 μmol, 5.00 equiv.), and tetrakis(triphenylphosphine)palladium (21.2 mg, 18.4 μmol, 10 mol‐%) were dissolved in 11.0 mL of degassed 10:1 THF/H_2_O. The solution was heated to 60 °C for 22 h. After cooling to rt, the aqueous layer was separated and the organic layer was precipitated into methanol and the product was collected via filtration. The crude product was further filtered through a short plug of aluminium oxide using EtOAc/petroleum ether (50:50, v/v) yielding the trimer (**9PP**_***anti,pre***_) as an off‐white solid (115 mg, 132 μmol, 72 %). ESI‐MS(+) (*m*/*z)*: calcd. for C_60_H_56_Br_2_O_2_ [Na+] 895.40, found 895.40, ^1^H NMR (300 MHz, CD_2_Cl_2_) δ [ppm]=7.79–7.25 (m, H_arom_., 26 H), 6.26–6.05 (m, H_olefin.,_ 12 H), 3.40–3.20 (m, ‐OMe, 18 H), ^13^C NMR: due to solubility issue not recordable. Elemental analysis calcd. for C_60_H_56_Br_2_O_2_: C=82.54; H=6.47; found: C=82.52; H=6.61: FTIR $\tilde{\nu }$ [cm^−1^]: 3087, 3052, 3030, 2933, 2900, 2822, 1600, 1491, 1465, 1446, 1400 1225, 1173, 1071, 1016, 949, 821, 761, 698.

## Conflict of interest

The authors declare no conflict of interest.

## Supporting information

As a service to our authors and readers, this journal provides supporting information supplied by the authors. Such materials are peer reviewed and may be re‐organized for online delivery, but are not copy‐edited or typeset. Technical support issues arising from supporting information (other than missing files) should be addressed to the authors.

Supplementary
