# Supplementary material for: Beyond p‐Hexaphenylenes: Synthesis of Unsubstituted p‐Nonaphenylene by a Precursor Protocol
Source: Chemistry. 2020 Dec 1;27(1):281–8. doi: 10.1002/chem.202001531 (PMC7839583; doi:10.1002/chem.202001531)
Supplement: Supplementary file 1 — Supplementary [file CHEM-27-281-s001.pdf]

Chemistry–A European Journal

Supporting Information

Beyond *p*-Hexaphenylenes: Synthesis of Unsubstituted *p*-Nonaphenylene by a Precursor Protocol

Ali Abdulkarim,[a, b] Marvin Nathusius,[a, b, e] Rainer Bäuerle,[b, c] Karl-Philipp Strunk,[c, d] Sebastian Beck,[b, c] Hans Joachim Räder,[e] Annemarie Pucci,[b, c, d] Christian Melzer,[b, c, d] Daniel Jänsch,[a, b] Jan Freudenberg,[a, b] Uwe H. F. Bunz,[a, b, d] and Klaus Müllen*[e]

Table of Content

1 General Remarks **1**

2 Annealing Process in Bulk and Thin Films **3**

2.1 Thermal Gravimetric Analysis (TGA) and Differential Scanning Calorimetry (DSC) 3

2.2 Dewetting Process of ***9PP**$_{syn,pre}$* 4

2.3 Polarization Microscopy 4

2.4 Absorption and Emission Spectra 6

2.5 Infrared Spectroscopy 9

3 Mass analysis of thin films **16**

4 Time-resolved photoluminescence **19**

5 X-ray Crystallographic Analysis **20**

6 NMR Spectra **24**

7 Solid-State NMR Spectra **28**

1 General Remarks

The ^{1}H NMR and ^{13}C NMR spectra were recorded on a Bruker AVANCE 300 and Bruker AVANCE 500. For calibration the residual solvent peak was referenced.[1]
Solid-state NMR CP/MAS experiments were performed with a Bruker AVANCE III console operating at 700 MHz 1H Lamor frequency using a commercial double resonance MAS probe supporting zirconia rotors with 2.5 mm outer diameter. The CP/MAS experiments were performed at 25 kHz MAS spinning frequency and ambient temperature with a CP contact time of 3ms if not stated differently in the figure caption. High-power frequency –swept TPPI hetero-nuclear dipolar decoupling of 100 kHz rf nutation frequency was applied during ^{13}C acquisition.
Thermogravimetric analysis and differential scanning calorimetry - analyses were done using a Mettler Toledo TGA/DSC1 device. Thin-films were made *via* spin-coating with a Spin Coater SCV-10 on quartz glass or Si-wafers. Layer-thicknesses of thin-films were determined using a profilometer (DektakXT, Bruker). Solid-state UV-Vis absorption and emission spectra were recorded at room temperature on a Jasco V660 and FP6500 spectrophotometer of thin-films on glass substrates. Microscope images were taken with a Nikon Eclipse LV100POL polarization microscope. AFM images were taken on a Bruker Nanoscope MultiMode VIII in the ScanAsyst PeakForce mode. The resulting AFM measurements were treated with the software Gwyddion for row alignment and scar correction. Melting points were determined on a Büchi B-545 hot stage apparatus.
Mass spectra (MS) were either recorded on the JEOL JMS-700 (EI+), Bruker ApexQehybrid 9.4 T FT-ICR-MS (ESI+), MALDI-TOF-MS spectra were reordered on a Bruker Daltonics Reflex III with *trans*-2-[3-(4-*tert*-Butylphenyl)-2-methyl-2-propenylidene] malononitrile (DCTB) as matrix or on a Bruker ApexQe hybrid 9.4 T FT-ICR. EI-MS experiments were carried out on Varian Saturn GC/MS 2000 or a Finnigan LCQ (ESI+) mass spectrometer at the Organic Chemical Institute of the University of Heidelberg.
Precursor solutions were spun-cast at 1000 rpm for 50 s in a nitrogen glove box with both water content and oxygen concentration below 3 ppm. Film thicknesses were determined via Vis ellipsometry and a Bruker DekTak XT profilometer. Films for mass analysis of **9PP** were prepared using drop coating and measured on a SynaptG2-Si using 7,7,8,8-tetracyanoquinodimethane (TCNQ) as matrix. The matrix was evaporated on the films using a heat plate under air. To ensure full aromatization of the thicker films

the films were heated at 300 °C for 5 h. Sample preparation: For all IR spectroscopic measurements, silicon wafers (1.5 x 1.5 cm^2, intrinsic, σ > 5000 Ω^{-1} cm^{-1}) with a native oxide layer were used as substrate. The substrates were cleaned prior to fabrication *via* sonication in acetone and isopropanol and dried using a dry nitrogen stream. Subsequently, the backside of the substrate was cleaned with $CHCl_3$. Thermal aromatization was achieved on standard thermal hotplates in nitrogen atmosphere inside the glovebox.

Infrared (IR)-Spectroscopy: All samples for IR transmission measurements were measured at room temperature using a Fourier-transform IR spectrometer (Vertex 80v) from Bruker. The spectrometer was evacuated to 3 mbar to prevent absorption from ambient air (water and CO_2). Spectra were recorded at near normal incidence (7 °) or – as specified – at 70° incident light. All spectra were recorded with an MCT detector, a resolution of 4 cm^{-1} and averaged over 200 scans.

Time resolved photoluminescence was determined on a Horiba FluoroCube-01-NL lifetime spectrofluorometer with emission monochromator.

The heating of the thin films was done stepwise in between measurements on a hotplate. After the specified time the sample was removed from the hotplate and put on a piece of aluminum at room temperature to prevent further heating and falsifying the time scale. Heating up and cooling down to under 50 °C took below 30 s, for 300 °C below 1 min.

Density functional theory (DFT) calculations were carried out with *Gaussian 09*[2] under use of the B3LYP[3-4] functional and the basis set 6-311G(d,p). To speed up this process a single reduced molecule (*p*-pentaphenylene) was calculated in gas phase. Simulations were carried out with SCOUT.[5]

Details of the crystal data and a summary of the intensity data collection parameters are listed at the end of this chapter. Suitable crystals were measured with Bruker Smart CCD diffractometer. Graphite-monochromated Mo K_α radiation was used. The structures were solved by direct methods with SIR-97 and refined by the full-matrix least-squares techniques against *F*2 (SHELXL-97). The intensities were corrected for Lorentz and polarization effects. The nonhydrogen atoms were refined anisotropically. Hydrogen atoms were refined using the riding model. The crystals structures were visualized using Mercury.

2 Annealing Process in Bulk and Thin Films

2.1 Thermal Gravimetric Analysis (TGA) and Differential Scanning Calorimetry (DSC)

The annealing process was studied by thermal gravimetric analysis (TGA) and differential scanning calorimetry (DSC) of the bulk precursor oligomers.

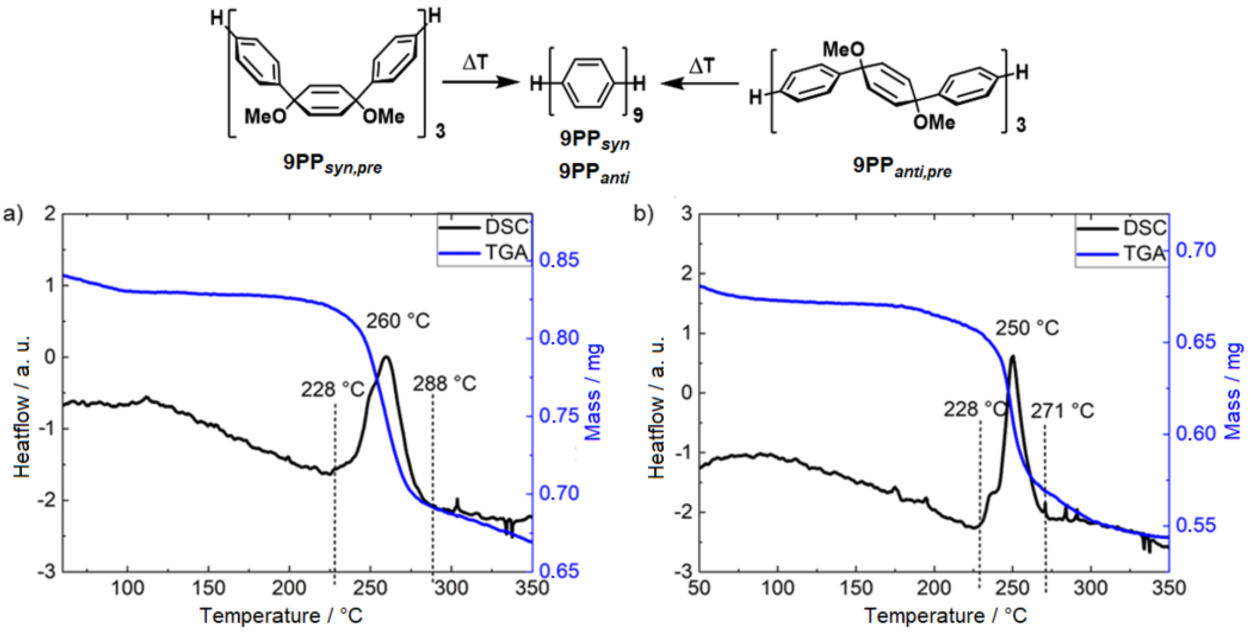

Figure S1: DSC and TGA-trace of **9PP**$_{syn,pre}$ (left) and **9PP**$_{anti,pre}$ (right) with a heating rate of 10 K/min. The TGA trace shows a loss in mass of the precursor oligomer at about 228-288 °C for the *syn*-isomer and 228-271 °C for the *anti*-isomer. This mass loss is attributed to demethoxylation to yield the corresponding phenylene units. Due to possible encapsulation of bulk material and the hindrance of conformational change from oligomer coils to rod-like oligomer the mass loss is slightly less than expected for both isomers (for syn-isomer: calc: 21.3%, observed: 18.2%; for *anti*-isomer: calc: 21.3%, observed: 20.0%;). However, the deviation of the expected vs. the observed mass loss for its polymeric analogues is much higher (calc: 21.38%, observed: 12.55%). Nevertheless, thin spin-coated thin films of the precursor materials (oligomer and polymer) show quantitative aromatization as evidenced by IR spectroscopy.

2.2 Dewetting Process of 9PP$_{syn,pre}$

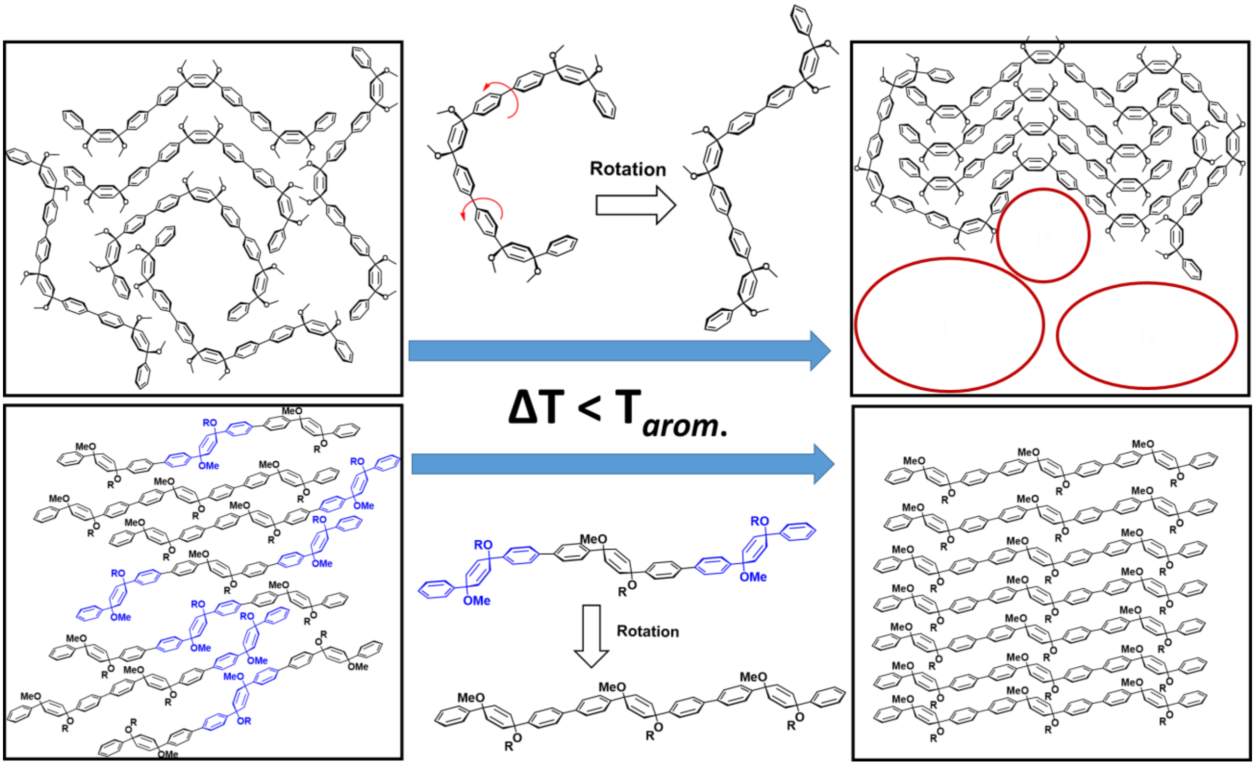

Figure S2: Illustration of the dewetting process as a result of the different shapes of the rotamers (*syn*- vs *anti*-isomer) and their impact on the packing. Heating below the aromatization temperature leads either to sole rearrangements (*anti*-isomer) or to dewetting (hole formation in the films as starting point for dewetting precess).[6-10]

2.3 Polarization Microscopy

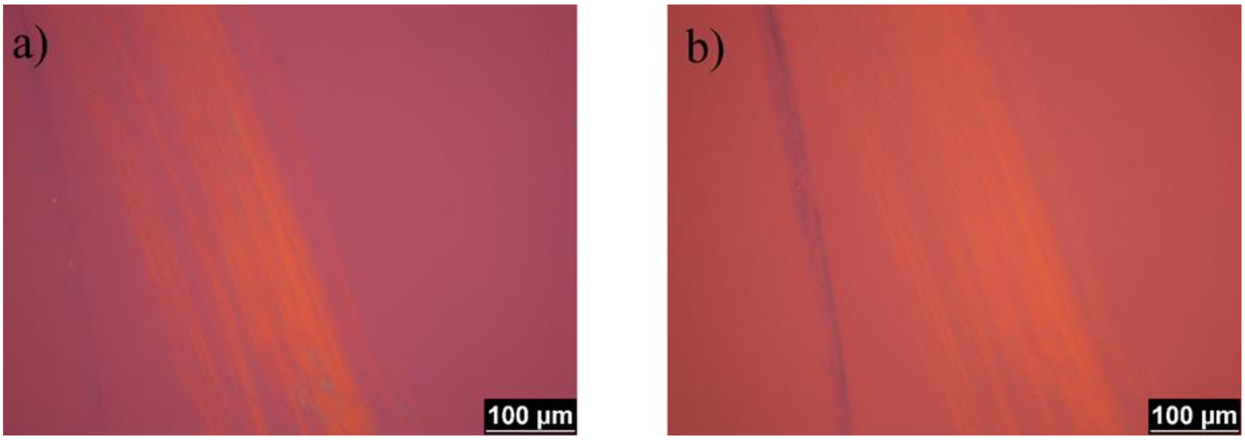

Figure S3: Polarization micrographs of in situ-heating **9PP**$_{anti,pre}$. Before heating (a) and after gradually heating to 300 °C (b) the film appear identical. Only a drift of the whole sample is visible due to the hardware of the experiment.

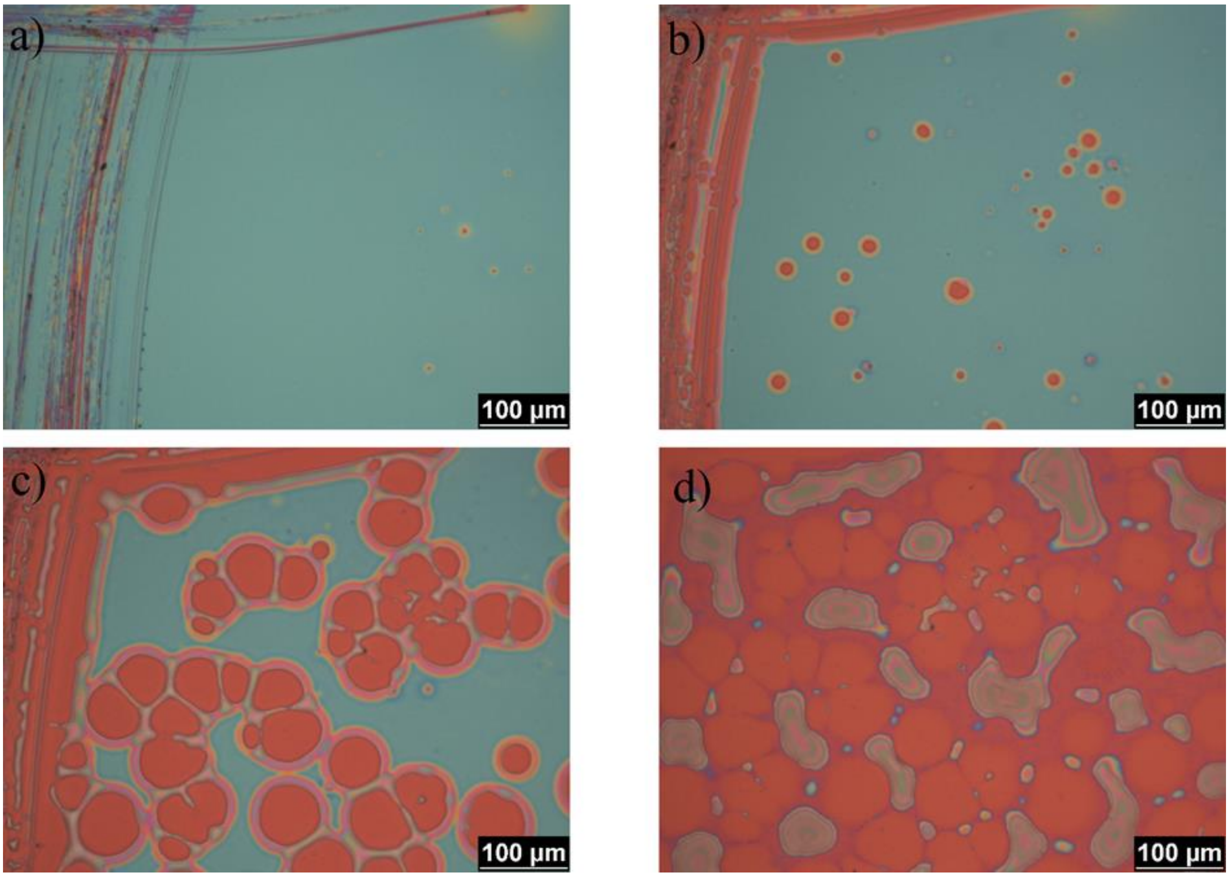

Figure S4: Polarization micrographs while heating **9PP**$_{syn, pre}$. Before heating (a) the film shows only small holes and a scratch used for focusing. At 150 °C (b) the dewetting has already started. At 175 °C (c) it is already quite severe and at 300 °C (d) only bubbles remain.

2.4 Absorption and Emission Spectra

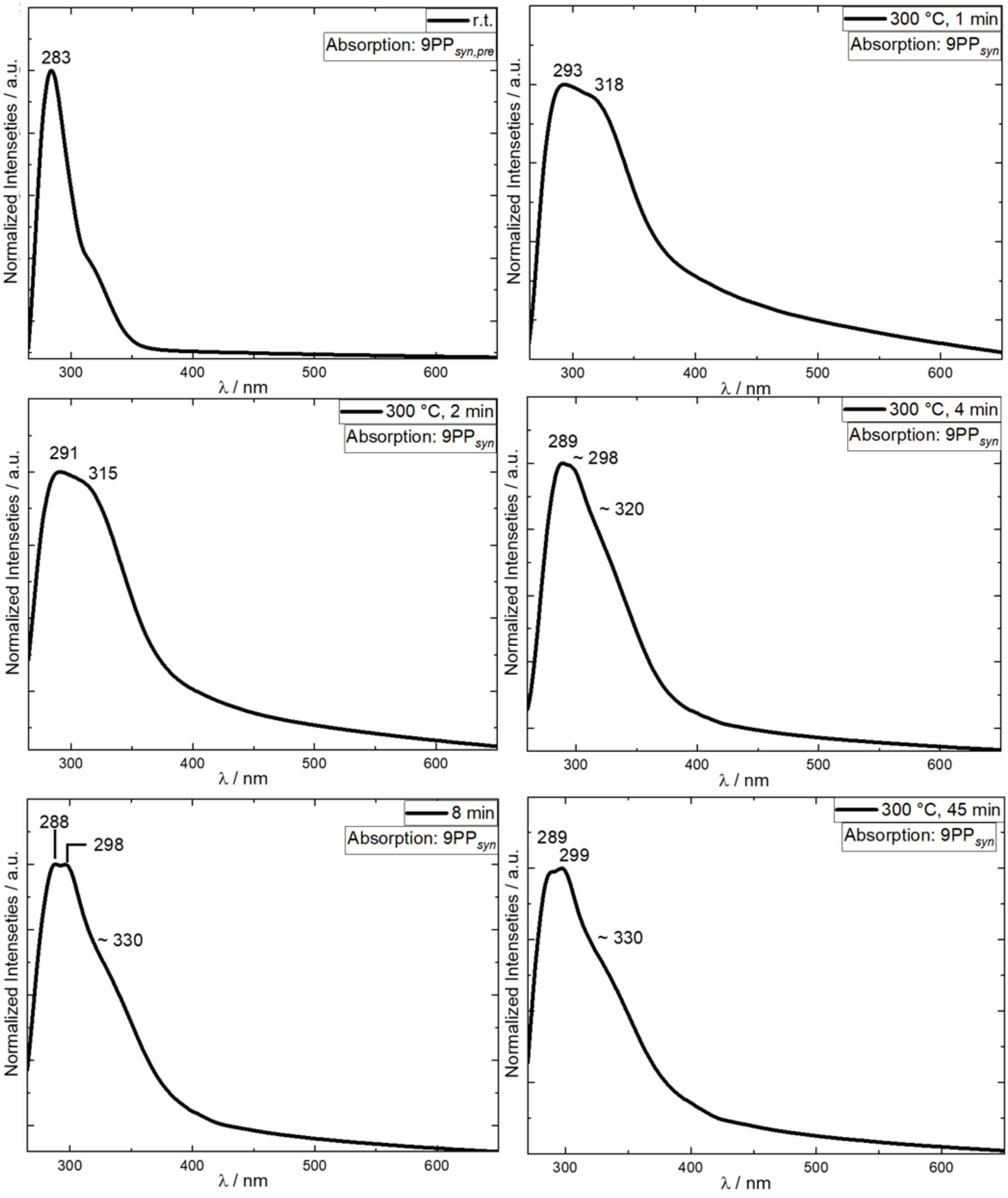

Figure S5: Time-dependent absorption spectra of **9PP**$_{syn,pre}$ annealed at 300 °C.

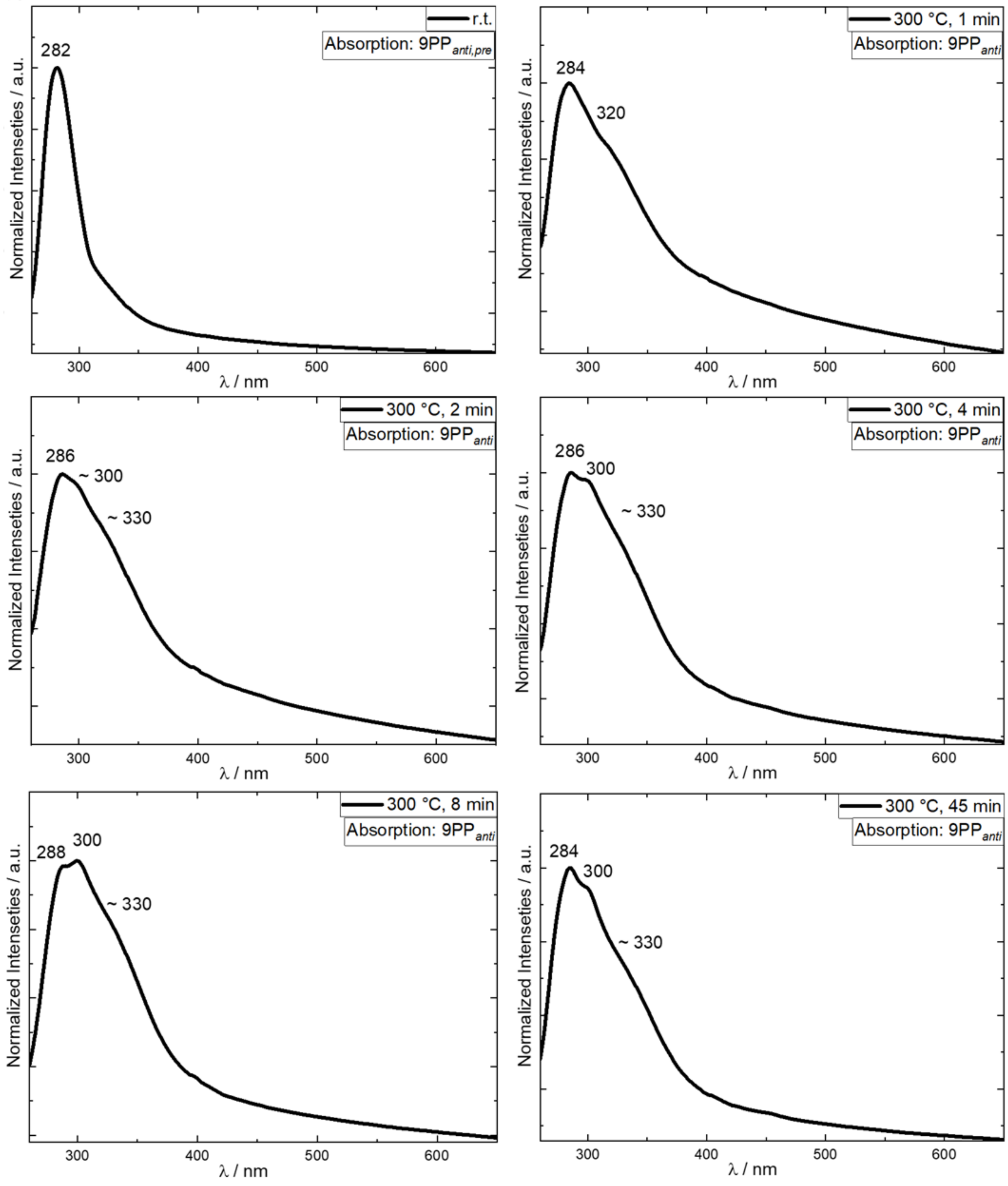

Figure S6: Time-dependent absorption spectra of **9PP*anti,pre*** annealed at 300 °C.

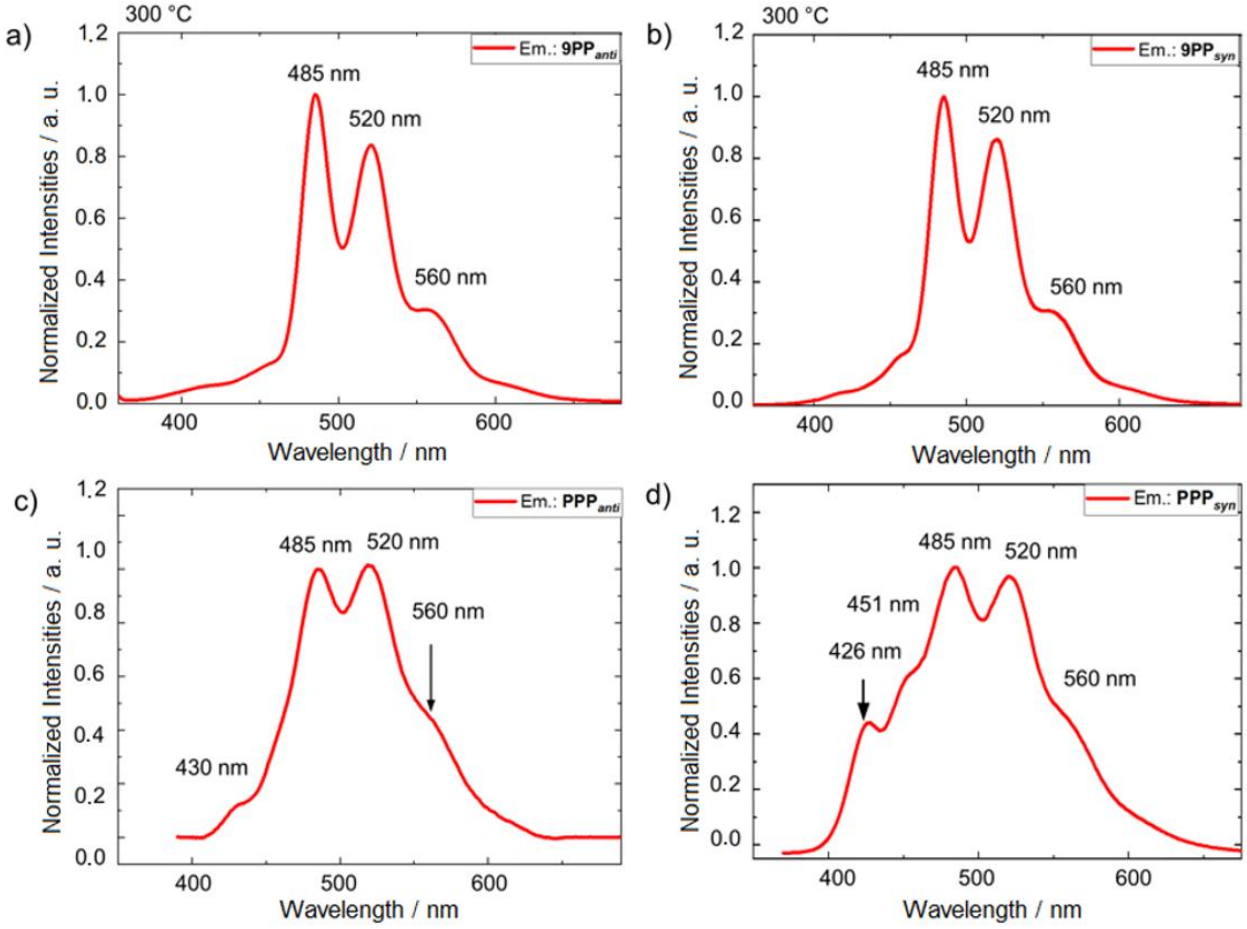

Figure S7: Emission spectra of the 9PPs (a: **9PP**$_{anti}$; b: **9PP**$_{syn}$) and PPPs (c: **PPP**$_{anti}$; d: **PPP**$_{syn}$).

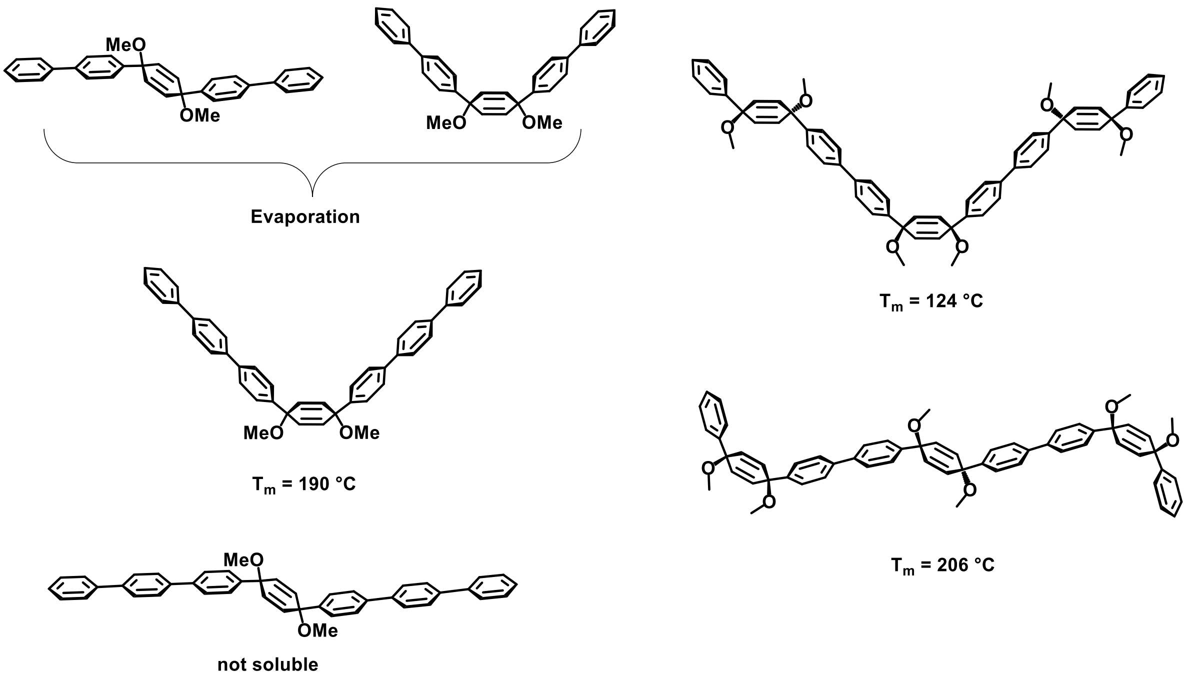

Figure S8: Further model compounds tested in our laboratories. T_m denotes respective melting temperatures.

2.5 Infrared Spectroscopy

While heating to 300 °C **9PP*anti,pre*** did keep a closed film wheras **9PP*syn,pre*** started dewetting at temperatures as low as 120 °C, see Figure S3 and Figure S4. Despite the dewetting of the films IR measurements were still possible because our IR beam is about 3 mm in diameter averaging the signal over islands and holes. This led to a wavy baseline and further artefacts in the spectra (Figure S12), which could still be used to roughly compare them to their **9PP*anti,pre*** counterparts and to sketch the evolution over time (Figure S13).

Table S1. Band assignment of **9PP*anti, pre***. δ and ν correspond to bending and stretching vibrations, respectively. The experimental wavelength ν_{exp} is compared with the predicted wavelength ν_{theo} from DFT calculations. From the latter the vibration can be assigned. Index "end" in the vibrational mode indicates that the vibration is mostly from the outermost phenyl ring, index "me" refers to vibrations only present in the methoxylated precursor and not in the converted **9PP*anti***. The orientation is given as in-plane (ip) or out-of-plane (oop) if assignment was possible.

Number Figure S9	ν_{exp} [cm^{-1}]	ν_{theo} [cm^{-1}]	Vibrational mode	Orientation
1	698	714	$\delta(C\text{-}H)_{end}$	ip
2	763	784	$\delta(C\text{-}H)_{end}$	
3	822	862	δ(C-H)	oop
4	951	967	$\nu(C\text{-}O)_{me}$	ip
5	1017	1029	ring deformation	ip
6	1072	1103	$\nu(C\text{-}O\text{-}C)_{me}$	ip
7	1175	1188	ν(C-C)	ip
8	1226	1238	ν(C-C)	ip
9	1400	1432	δ(C-H)	ip
10	1491	1520	δ(C-H)	ip
11	2822	2999	$\nu(C\text{-}H_3)_{me}$	ip

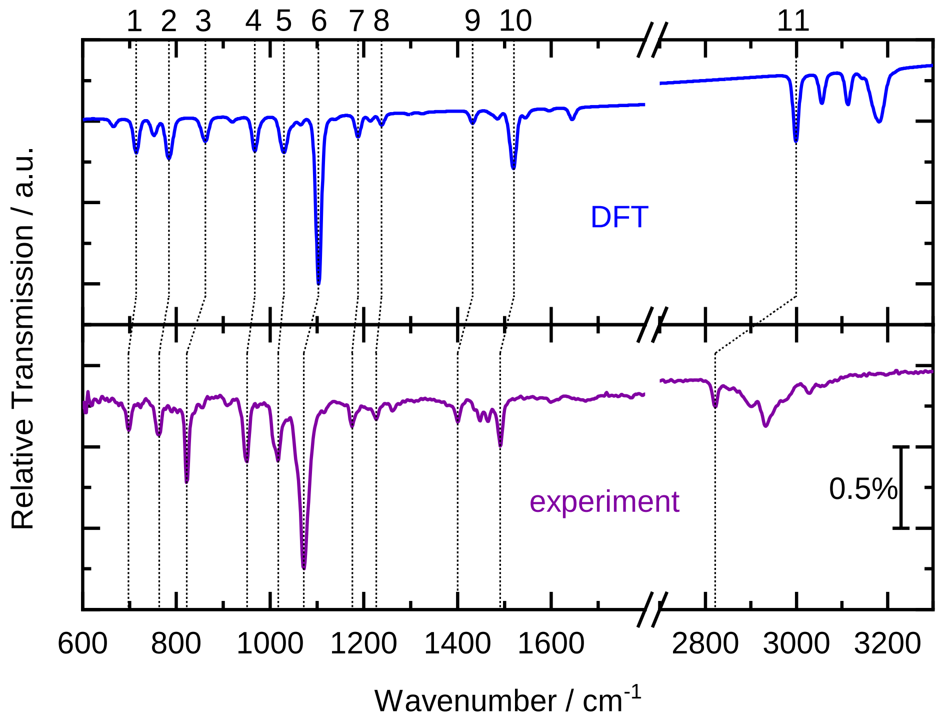

Figure S9: DFT calculated and simulated IR transmission spectrum (top) and experimental (bottom) spectrum of **9PP**$_{anti,\ pre}$. The numbered lines indicate the assigned band vibrations in Table S1.

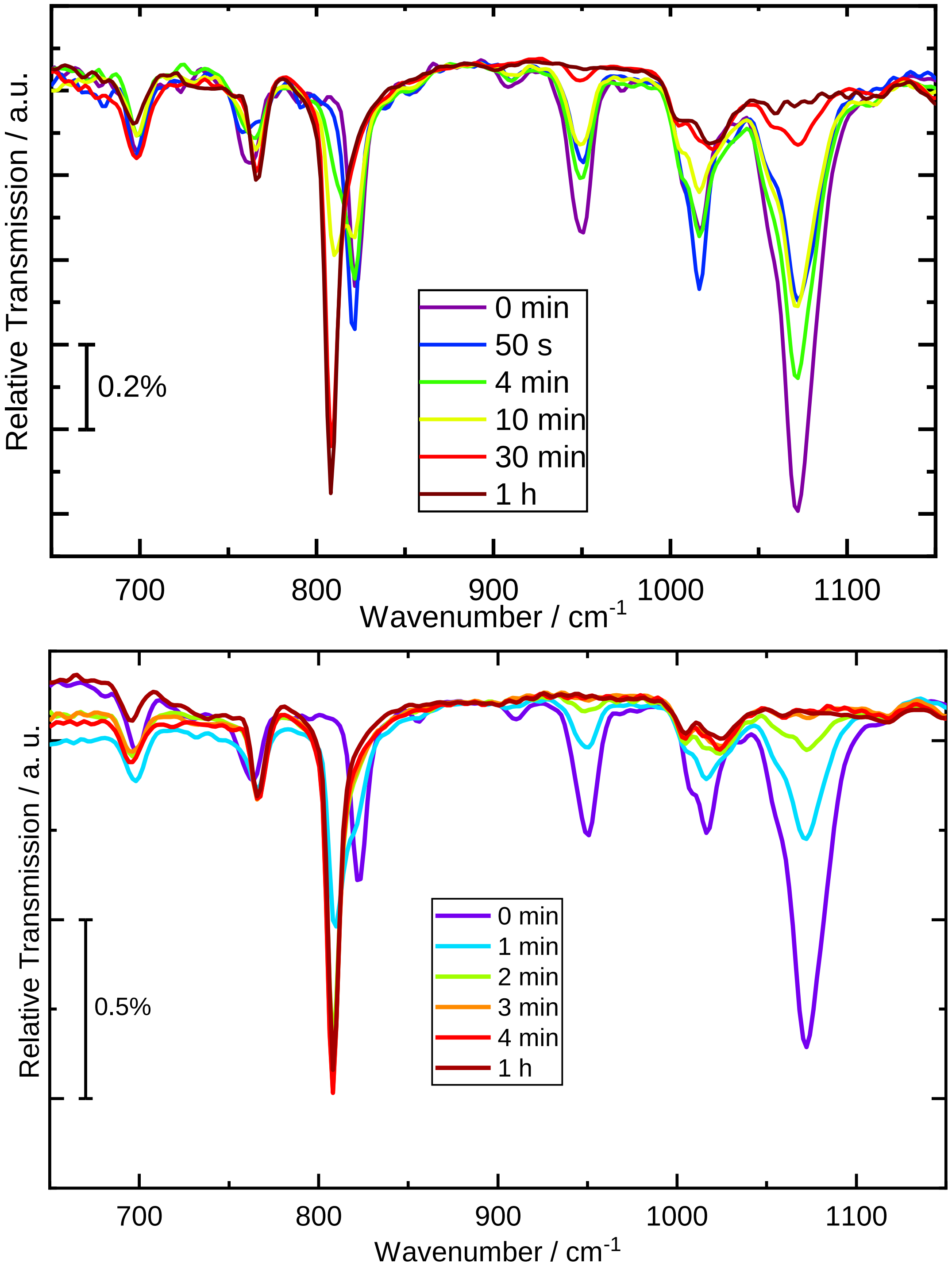

Figure S10: Time-resolved infrared spectroscopy of **9PP***anti, pre* annealed at 250 °C (top) and 300 °C (bottom). The precursor specific absorption bands (e.g. 950 cm^{-1} or 1076 cm^{-1}) gradually decrease with time, at 250 °C at a slower rate than at 300 °C.

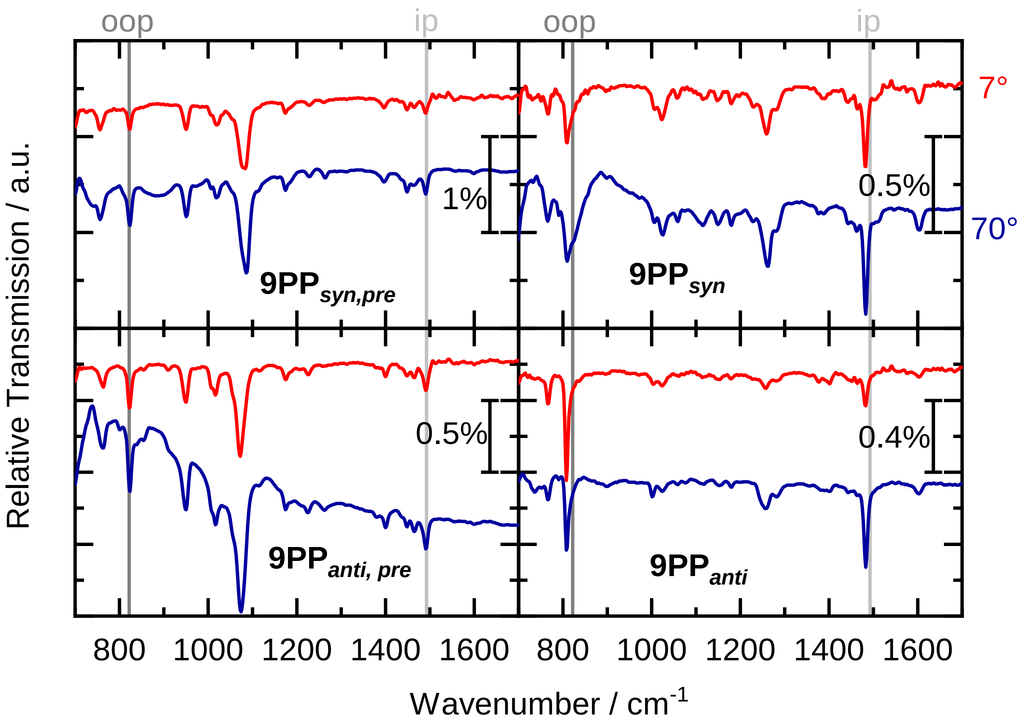

Figure S11: FTIR spectra of **9PP**$_{syn,\ pre}$, **9PP**$_{syn}$, **9PP**$_{anti,\ pre}$ and **9PP**$_{anti}$ under normal (red) and flat (green) incidence. The difference of the intensity ratio of the out-of-plane (oop, dark grey) and the in-plane (ip, light grey) vibrations indicates the different preferred molecular orientation within the film. The orientations found are face-on for **9PP**$_{syn,\ pre}$, face-on for **9PP**$_{anti,\ pre}$ and edge-on for **9PP**$_{anti}$. The dewetted **9PP**$_{syn}$ did not exhibit a preferred molecular orientation.

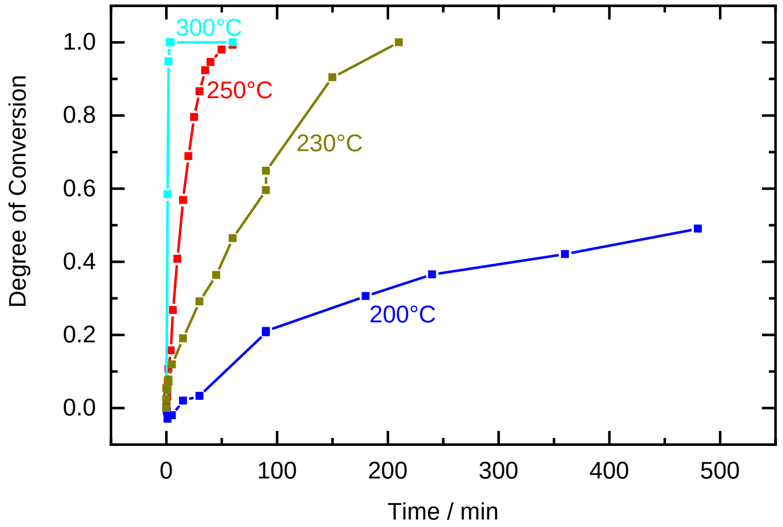

Figure S12: Degree of conversion from **9PP**$_{anti,\ pre}$ to **9PP**$_{anti}$ at 300 °C (cyan), 250 °C (red), 230 °C (gold) and 200 °C (blue). Due to the long time the 200 °C series was aborted halfway through the aromatization. Each point corresponds to one measurement.

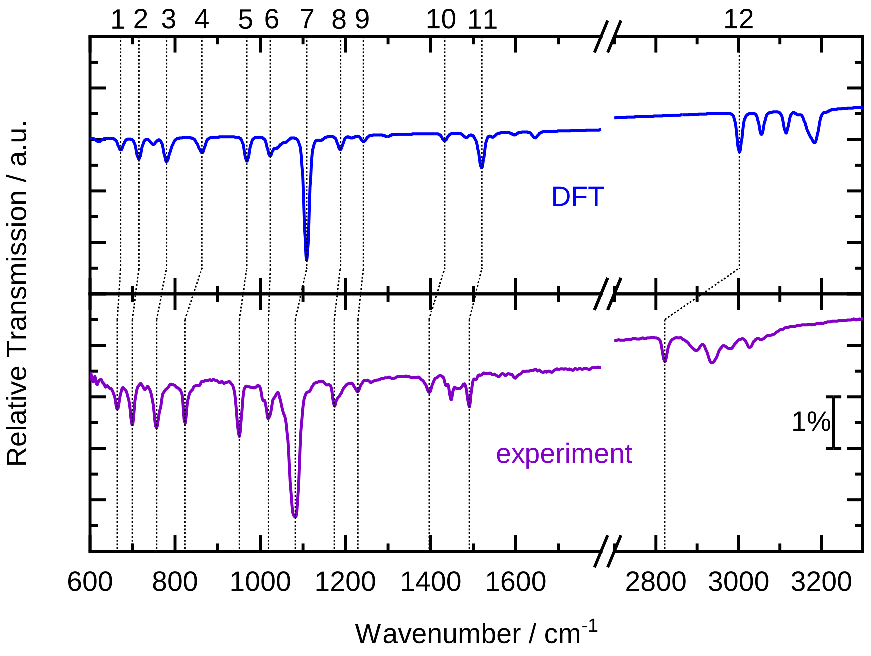

Figure S13: DFT calculated and simulated IR transmission spectrum (top) and experimental (bottom) spectrum of **9PP*syn, pre***. The numbered lines indicate the assigned band vibrations in Table S2.

Table S2: Band assignment of **9PP*syn, pre***. The nomenclature follows that of Table S1.

Number Figure S13	ν_{exp} [cm^{-1}]	ν_{theo} [cm^{-1}]	Vibrational mode	Orientation
1	664	672	$\nu(C\text{-}O)_{me}$	ip
2	699	715	$\delta(C\text{-}H)_{end}$	oop
3	756	779	$\delta(C\text{-}H)$	oop
4	823	864	$\delta(C\text{-}H)$	oop
5	951	968	$\nu(C\text{-}O)_{me}$	ip
6	1019	1023	$\delta(C\text{-}H)_{me}$	ip
7	1082	1109	$\nu(C\text{-}O\text{-}C)_{me}$	ip
8	1174	1188	$\nu(C\text{-}C)$	ip
9	1229	1242	$\nu(C\text{-}C)$	ip
10	1396	1433	$\delta(C\text{-}H)$	ip
11	1491	1520	$\delta(C\text{-}H)$	ip
12	2822	3002	$\nu(C\text{-}H_3)_{me}$	ip

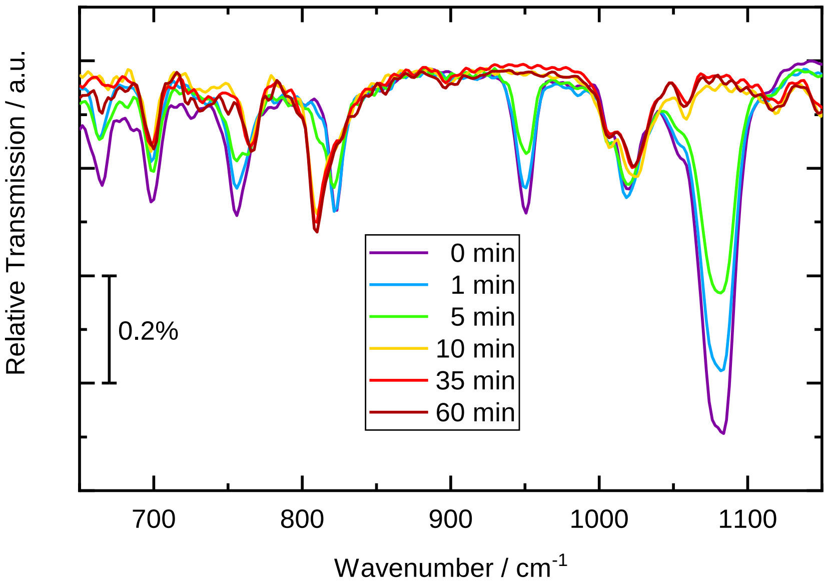

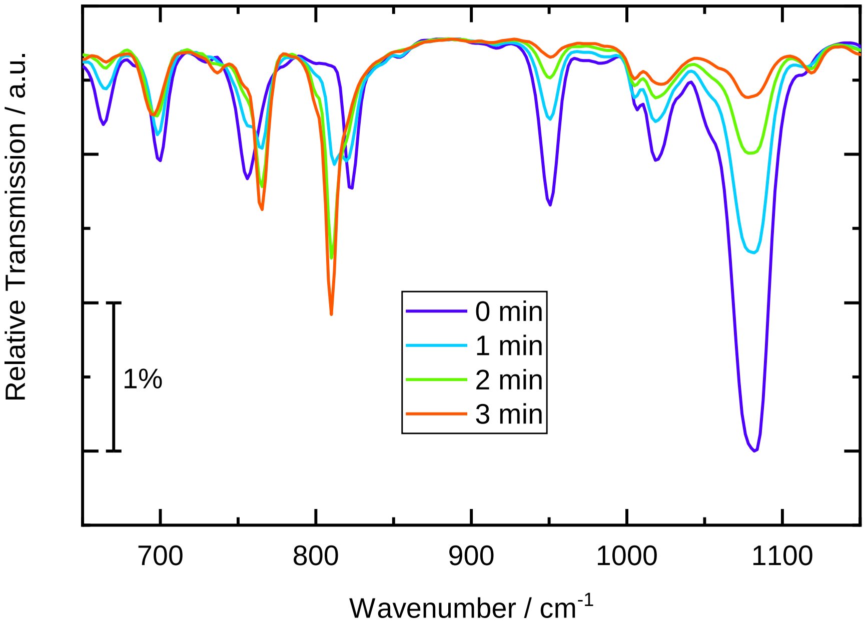

Figure S14: Time-resolved infrared spectroscopy of **9PP**$_{\mathit{syn,\ pre}}$ annealed at 250 °C (top) and 300 °C (bottom). As for **9PP**$_{\mathit{anti,\ pre}}$ precursor specific absorption bands gradually decrease. Due to dewetting IR-measurement was stopped after 3 min.

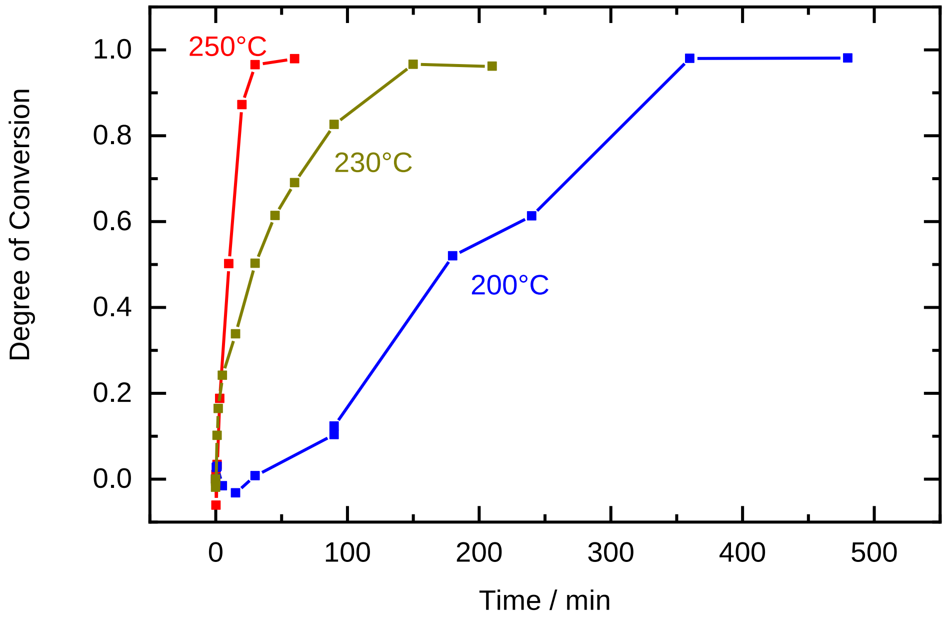

Figure S15: Degree of conversion from **9PP**$_{syn,\ pre}$ to **9PP**$_{syn}$ at different temperatures. Dewetting leads to distortion especially at the beginning.

3 Mass analysis of thin films

Drop-coated films (2 mg/mL, chloroform) were prepared using **9PP*anti,pre*** as precursor. The films were thermally annealed at 300 °C and coated with a thin layer of TCNQ (7,7,8,8-tetracyanoquinodimethane) as a MALDI matrix. Due to aggregation effects of **9PP** molecules, high laser intensities were needed for desorption during mass analysis, also resulting in partial fragmentation during the measurement, the ratio of which was highly dependent on the exact spot used for desorption. Under the same conditions, analysis of **9PP*anti,pre*** films was unsuccessful - measurements resulted in fragmentation, some of the observed m/z ratios being identical to peaks observed for analysis of the **9PP*anti*** films ,but no precursor molecular peak was found. Thus, we conclude that **9PP** is indeed formed with our thermal aromatization procedure.

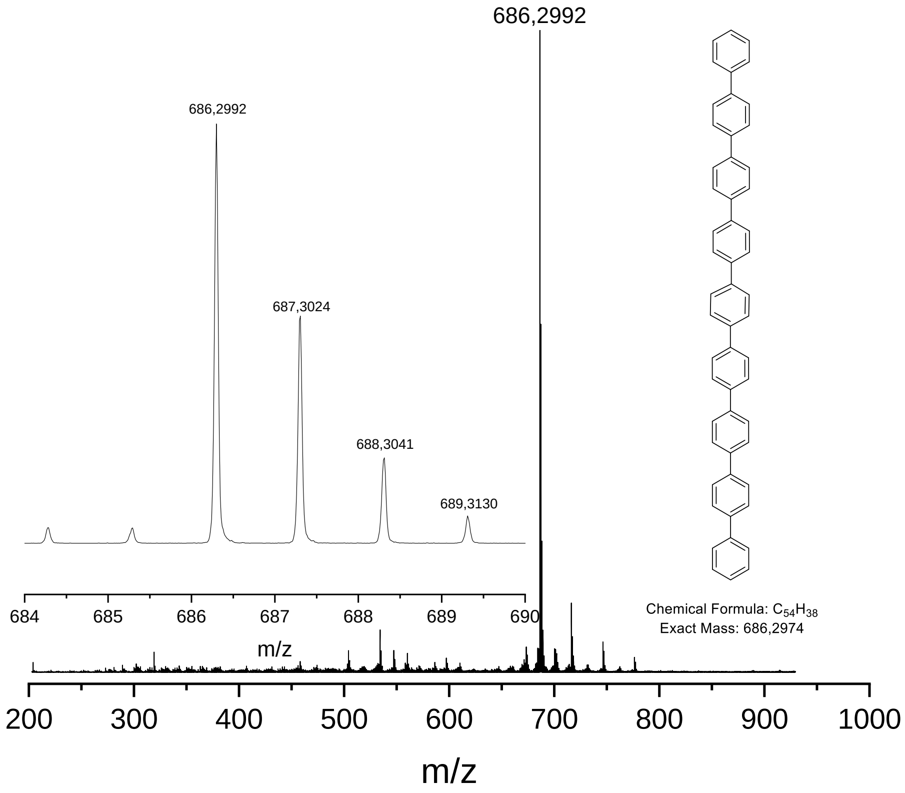

Figure S16: MALDI spectra of **9PP*anti*** film using TCNQ as matrix.

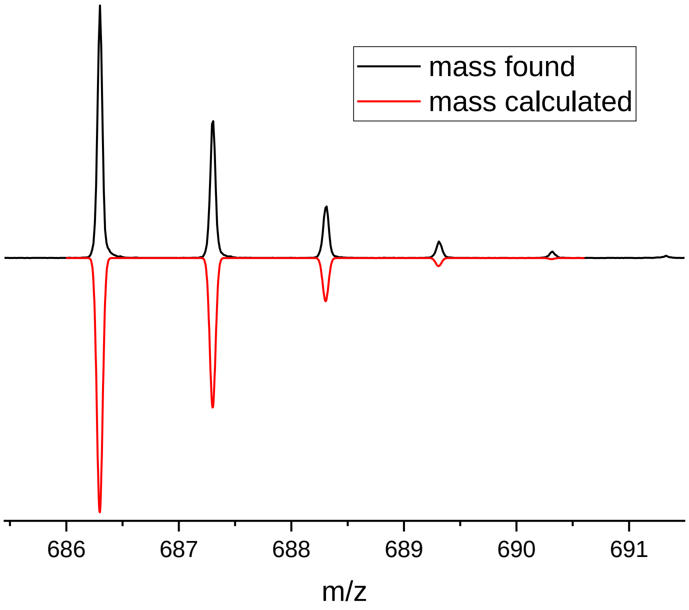

Figure S17: Measured isotope pattern of **9PP*anti*** (black) compared to calculated isotope pattern (red).

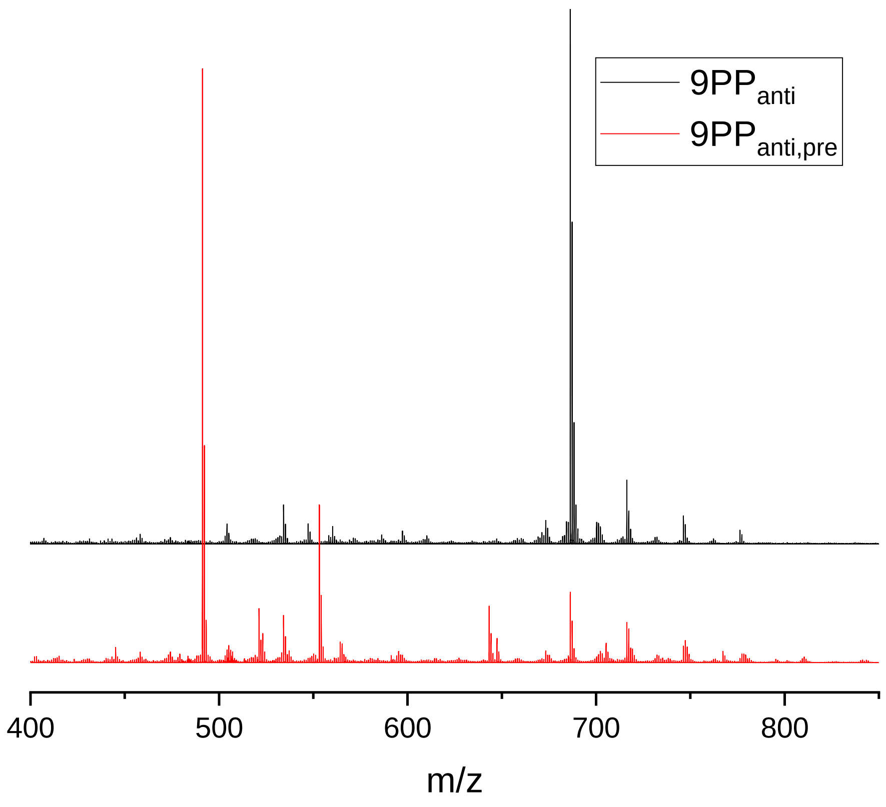

Figure S18: MALDI-spectra of **9PP*anti*** (black) compared to mass spectra of **9PP*anti,pre*** (red) showing similar fragmentation patterns at around 700 m/z. Peaks observed at higher m/z ratios for **9PP** do not match exactly any partially aromatized **9PP** precursor structures (indicating incomplete conversion), as m/z ratios are by 2 m/z too low as, e.g. a fourfold demethoxylated precursor. Intensities from the spectrum cannot be regarded as a measure of purity due to the desorption problems of the fully aromatized rigid-rod-like hydrocarbon product.

4 Time-resolved photoluminescence

Table S3: Emission lifetimes of **9PP**$_{\textbf{\textit{anti/syn}}}$ thermally generated in thin films.

Film	Excitation Wavelength nm	Lifetime 1 ns/%	Lifetime 2 ns/%	Lifetime 3 ns/%
**9PP**$_{\textbf{\textit{anti}}}$	430	0.2/34	0.4/58	0.7/8
	485	3.1/43	6.1/44	12.3/13
	520	3.1/44	6.3/51	12.5/5
	560	2.7/39	5.4/32	10.8/29
**9PP**$_{\textbf{\textit{syn}}}$	430	0.3/41	0.6/48	1.2/11
	485	2.9/43	5.8/44	11.5/13
	520	2.6/46	5.2/44	10.4/10
	560	3.2/44	6.5/19	13.1/37

Lifetimes were fitted with a tris-exponential function. While the emission maxima at 485 nm, 520 nm and 560 nm show nearly the same lifetimes and fractions (both **9PP**$_{\textbf{\textit{anti}}}$ and **9PP**$_{\textbf{\textit{syn}}}$), the lifetimes for the weak emission at 430 nm are significantly shorter. In accordance with literature,[11,12] emission bands at 485, 520, 560 nm can be assigned to aggregates and the one at 430 nm to another species, most likely resulting from single-strand emission, as in thin-films more than one emitting species is likely to be present. The same trend was previously found for *para*-hexaphenylen.[13,14]

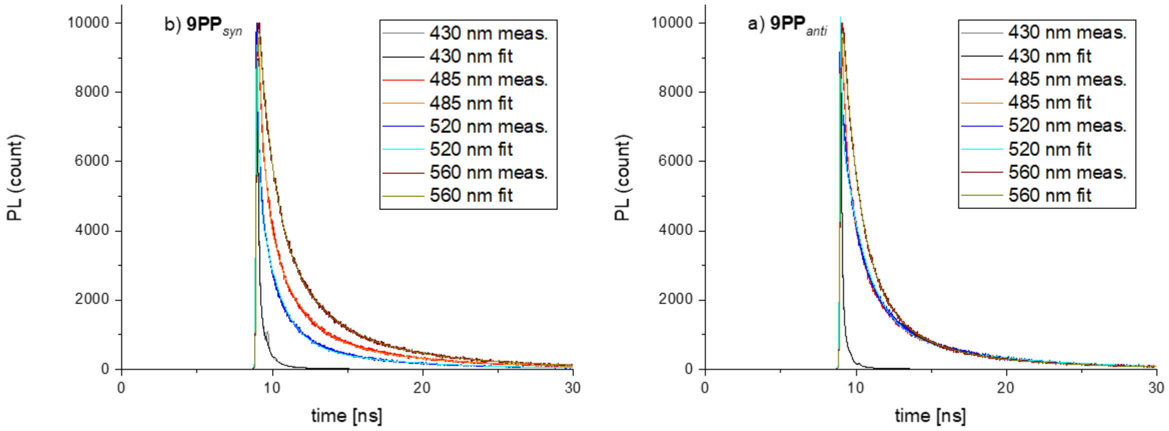

Figure S19: Lifetime measurements of **9PP**$_{\textbf{\textit{anti/syn}}}$ in thin film.

5 X-ray Crystallographic Analysis

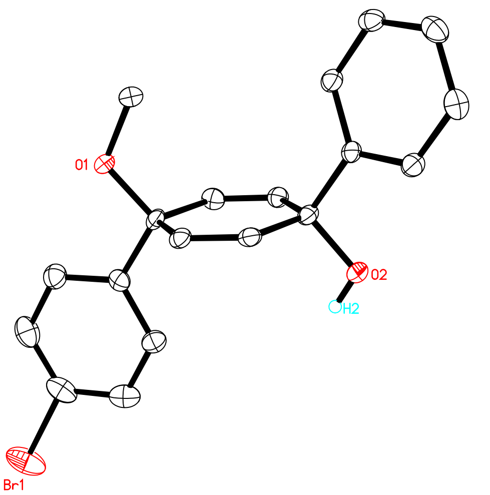

Figure S20: Crystal structure of ***anti*-2** (CCDC: 1981192).

Table S4: Crystal data and structure refinement.

Empirical formula	$C_{19}H_{17}BrO_2$	
Formula weight	357.23	
Temperature	100(2) K	
Wavelength	1.54178 Å	
Crystal system	monoclinic	
Space group	$P2_1/c$	
Z	4	
Unit cell dimensions	a = 19.153(6) Å	α = 90 deg.
	b =7.2001(15) Å	β =95.86(3) deg.
	c = 11.743(4) Å	γ = 90 deg.
Volume	1610.9(8) $Å^3$	
Density (calculated)	1.47 g/cm^3	
Absorption coefficient	3.51 mm^{-1}	
Crystal shape	brick	
Crystal size	0.181 x 0.120 x 0.053 mm^3	
Crystal colour	colourless	
Theta range for data collection	4.6 to 68.5 deg.	
Index ranges	-22≤h≤22, -8≤k≤3, -14≤l≤13	
Reflections collected	7548	
Independent reflections	2858 (R(int) = 0.0193)	
Observed reflections	2606 (I > 2σ(I))	
Absorption correction	Semi-empirical from equivalents	
Max. and min. transmission	1.71 and 0.46	
Refinement method	Full-matrix least-squares on F^2	
Data/restraints/parameters	2858 / 0 / 204	
Goodness-of-fit on F^2	1.04	
Final R indices (I>2sigma(I))	R1 = 0.044, wR2 = 0.105	
Largest diff. peak and hole	1.88 and -1.39 $eÅ^{-3}$	

Table S5: Atomic coordinates and equivalent isotropic displacement parameters ($Å^2$) . U_{eq} is defined as one third of the trace of the orthogonalized U_{ij} tensor.

Atom	x	y	z	U_{eq}
Br1	0.0168(1)	1.2851(1)	0.6762(1)	0.0466(2)
O1	0.2458(1)	0.6396(3)	0.5141(2)	0.0190(4)
O2	0.3105(1)	0.5166(3)	0.9557(2)	0.0226(5)
H2	0.287(2)	0.606(6)	0.959(3)	0.033(11)
C11	0.2384(1)	0.6695(4)	0.6344(2)	0.0166(6)
C12	0.3072(1)	0.7387(4)	0.6930(2)	0.0167(6)
H12	0.3268	0.8478	0.6637	0.020
C13	0.3421(2)	0.6574(4)	0.7825(2)	0.0171(6)
H13	0.3845	0.7134	0.8145	0.021
C14	0.3187(1)	0.4821(4)	0.8368(2)	0.0171(6)
C15	0.2500(2)	0.4129(4)	0.7781(2)	0.0182(6)
H15	0.2306	0.3034	0.8072	0.022
C16	0.2147(2)	0.4940(4)	0.6886(2)	0.0191(6)
H16	0.1721	0.4383	0.6569	0.023
C18	0.2938(2)	0.4939(4)	0.4924(2)	0.0238(6)
H18A	0.3011	0.4935	0.4110	0.036
H18B	0.2743	0.3740	0.5129	0.036
H18C	0.3388	0.5146	0.5384	0.036
C21	0.1827(1)	0.8195(4)	0.6394(2)	0.0178(6)
C22	0.1436(2)	0.8898(4)	0.5425(3)	0.0251(6)
H22	0.1506	0.8425	0.4690	0.030
C23	0.0944(2)	1.0294(5)	0.5531(3)	0.0311(7)
H23	0.0678	1.0777	0.4870	0.037
C24	0.0844(2)	1.0971(4)	0.6598(3)	0.0297(7)
C25	0.1221(2)	1.0283(4)	0.7571(3)	0.0277(7)
H25	0.1147	1.0755	0.8305	0.033
C26	0.1709(2)	0.8897(4)	0.7461(2)	0.0222(6)
H26	0.1968	0.8414	0.8128	0.027
C31	0.3771(2)	0.3385(4)	0.8362(2)	0.0167(6)
C32	0.4315(2)	0.3370(4)	0.9244(2)	0.0207(6)
H32	0.4307	0.4220	0.9861	0.025
C33	0.4868(2)	0.2133(4)	0.9233(3)	0.0243(6)
H33	0.5233	0.2132	0.9845	0.029
C34	0.4891(2)	0.0896(4)	0.8335(3)	0.0239(6)
H34	0.5271	0.0049	0.8325	0.029
C35	0.4353(2)	0.0911(4)	0.7451(2)	0.0215(6)
H35	0.4364	0.0065	0.6833	0.026
C36	0.3798(2)	0.2146(4)	0.7458(2)	0.0198(6)
H36	0.3434	0.2146	0.6844	0.024

Table S6: Hydrogen coordinates and isotropic displacement parameters (Å^2).

Atom	x	y	z	U_{eq}
H2	0.287(2)	0.606(6)	0.959(3)	0.033(11)
H12	0.3268	0.8478	0.6637	0.020
H13	0.3845	0.7134	0.8145	0.021
H15	0.2306	0.3034	0.8072	0.022
H16	0.1721	0.4383	0.6569	0.023
H18A	0.3011	0.4935	0.4110	0.036
H18B	0.2743	0.3740	0.5129	0.036
H18C	0.3388	0.5146	0.5384	0.036
H22	0.1506	0.8425	0.4690	0.030
H23	0.0678	1.0777	0.4870	0.037
H25	0.1147	1.0755	0.8305	0.033
H26	0.1968	0.8414	0.8128	0.027
H32	0.4307	0.4220	0.9861	0.025
H33	0.5233	0.2132	0.9845	0.029
H34	0.5271	0.0049	0.8325	0.029
H35	0.4364	0.0065	0.6833	0.026
H36	0.3434	0.2146	0.6844	0.024

Table S7: Anisotropic displacement parameters (Å^2) for ala24. The anisotropic displacement factor exponent takes the form: $-2 pi^2 (h^2 a^{*2} U_{11} + ... + 2 h k a^* b^* U_{12})$

Atom	U_{11}	U_{22}	U_{33}	U_{23}	U_{13}	U_{12}
Br1	0.0284(2)	0.0331(2)	0.0781(3)	-0.0104(2)	0.0046(2)	0.0110(2)
O1	0.0258(10)	0.0190(10)	0.0124(9)	-0.0011(8)	0.0032(7)	0.0031(8)
O2	0.0312(12)	0.0238(11)	0.0134(10)	0.0000(8)	0.0053(8)	0.0077(10)
C11	0.0203(14)	0.0193(13)	0.0104(12)	0.0003(10)	0.0026(10)	-0.0013(11)
C12	0.0205(14)	0.0161(13)	0.0143(13)	-0.0008(10)	0.0058(10)	-0.0025(11)
C13	0.0210(13)	0.0151(13)	0.0159(13)	-0.0035(10)	0.0047(10)	-0.0011(11)
C14	0.0204(14)	0.0187(14)	0.0127(13)	-0.0012(10)	0.0040(10)	0.0001(11)
C15	0.0216(14)	0.0164(13)	0.0175(13)	0.0010(11)	0.0068(11)	-0.0007(11)
C16	0.0180(13)	0.0171(14)	0.0226(14)	-0.0008(11)	0.0034(11)	-0.0026(11)
C18	0.0320(16)	0.0215(15)	0.0185(14)	-0.0024(11)	0.0055(12)	0.0058(13)
C21	0.0171(13)	0.0153(13)	0.0214(14)	0.0011(11)	0.0035(11)	-0.0028(11)
C22	0.0239(15)	0.0284(16)	0.0232(15)	0.0022(12)	0.0040(12)	0.0028(13)
C23	0.0224(15)	0.0313(18)	0.0387(19)	0.0087(15)	-0.0012(13)	0.0039(13)
C24	0.0197(15)	0.0208(15)	0.049(2)	-0.0014(14)	0.0057(14)	0.0000(12)
C25	0.0223(15)	0.0263(16)	0.0356(18)	-0.0083(13)	0.0080(13)	-0.0047(13)
C26	0.0214(14)	0.0244(15)	0.0212(14)	-0.0018(12)	0.0045(11)	-0.0009(12)
C31	0.0231(14)	0.0142(13)	0.0136(13)	0.0027(10)	0.0056(10)	-0.0007(11)
C32	0.0264(15)	0.0188(14)	0.0168(14)	-0.0005(11)	0.0024(11)	-0.0001(12)
C33	0.0238(15)	0.0235(15)	0.0252(16)	0.0050(12)	0.0004(12)	0.0000(12)
C34	0.0239(15)	0.0186(15)	0.0301(16)	0.0049(12)	0.0080(12)	0.0031(12)
C35	0.0281(15)	0.0177(14)	0.0201(14)	-0.0006(11)	0.0098(12)	-0.0015(12)
C36	0.0260(15)	0.0182(14)	0.0153(14)	0.0007(11)	0.0034(11)	-0.0007(11)

Table S8: Bond lengths (Å) and angles (deg).

Br1-C24	1.897(3)	O2-C14-C13	109.4(2)
O1-C18	1.435(3)	O2-C14-C15	108.9(2)
O1-C11	1.450(3)	C13-C14-C15	111.6(2)
O2-C14	1.443(3)	O2-C14-C31	105.9(2)
O2-H2	0.79(4)	C13-C14-C31	108.5(2)
C11-C16	1.506(4)	C15-C14-C31	112.4(2)
C11-C12	1.508(4)	C16-C15-C14	124.6(3)
C11-C21	1.523(4)	C16-C15-H15	117.7
C12-C13	1.323(4)	C14-C15-H15	117.7
C12-H12	0.9500	C15-C16-C11	123.6(3)
C13-C14	1.503(4)	C15-C16-H16	118.2
C13-H13	0.9500	C11-C16-H16	118.2
C14-C15	1.507(4)	O1-C18-H18A	109.5
C14-C31	1.524(4)	O1-C18-H18B	109.5
C15-C16	1.325(4)	H18A-C18-H18B	109.5
C15-H15	0.9500	O1-C18-H18C	109.5
C16-H16	0.9500	H18A-C18-H18C	109.5
C18-H18A	0.9800	H18B-C18-H18C	109.5
C18-H18B	0.9800	C22-C21-C26	118.7(3)
C18-H18C	0.9800	C22-C21-C11	123.2(3)
C21-C22	1.392(4)	C26-C21-C11	118.1(2)
C21-C26	1.392(4)	C21-C22-C23	120.2(3)
C22-C23	1.393(4)	C21-C22-H22	119.9
C22-H22	0.9500	C23-C22-H22	119.9
C23-C24	1.375(5)	C24-C23-C22	119.7(3)
C23-H23	0.9500	C24-C23-H23	120.2
C24-C25	1.381(5)	C22-C23-H23	120.2
C25-C26	1.381(4)	C23-C24-C25	121.1(3)
C25-H25	0.9500	C23-C24-Br1	120.3(2)
C26-H26	0.9500	C25-C24-Br1	118.5(2)
C31-C36	1.391(4)	C26-C25-C24	118.9(3)
C31-C32	1.392(4)	C26-C25-H25	120.5
C32-C33	1.386(4)	C24-C25-H25	120.5
C32-H32	0.9500	C25-C26-C21	121.4(3)
C33-C34	1.384(4)	C25-C26-H26	119.3
C33-H33	0.9500	C21-C26-H26	119.3
C34-C35	1.387(4)	C36-C31-C32	118.7(3)
C34-H34	0.9500	C36-C31-C14	121.5(2)
C35-C36	1.387(4)	C32-C31-C14	119.7(2)
C35-H35	0.9500	C33-C32-C31	120.9(3)
C36-H36	0.9500	C33-C32-H32	119.6
C18-O1-C11	114.2(2)	C31-C32-H32	119.6
C14-O2-H2	108(3)	C34-C33-C32	120.3(3)
O1-C11-C16	110.6(2)	C34-C33-H33	119.9
O1-C11-C12	109.0(2)	C32-C33-H33	119.9
C16-C11-C12	111.8(2)	C33-C34-C35	119.1(3)
O1-C11-C21	106.3(2)	C33-C34-H34	120.5
C16-C11-C21	109.6(2)	C35-C34-H34	120.5
C12-C11-C21	109.3(2)	C36-C35-C34	120.8(3)
C13-C12-C11	124.2(3)	C36-C35-H35	119.6
C13-C12-H12	117.9	C34-C35-H35	119.6
C11-C12-H12	117.9	C35-C36-C31	120.3(3)
C12-C13-C14	124.1(3)	C35-C36-H36	119.9
C12-C13-H13	117.9	C31-C36-H36	119.9
C14-C13-H13	117.9		

6 NMR Spectra

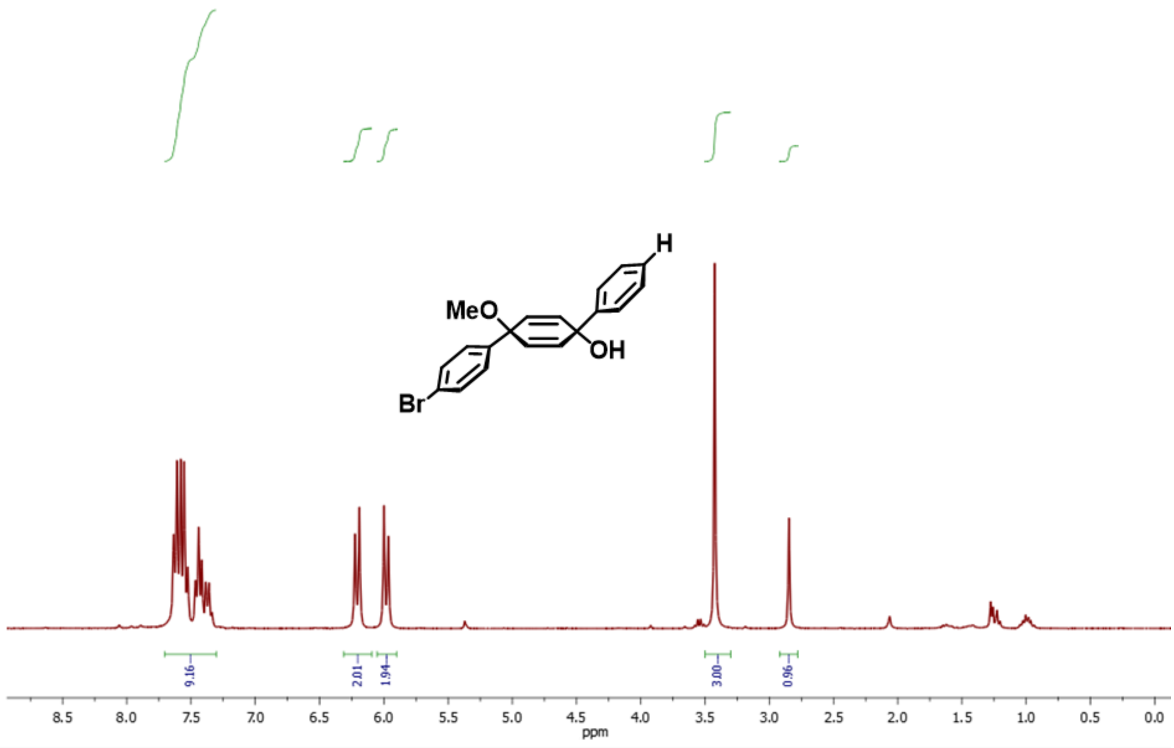

Figure S21: ^{1}H NMR spectrum of ***anti*-2** in CD_2Cl_2 (300 MHz, 298 K).

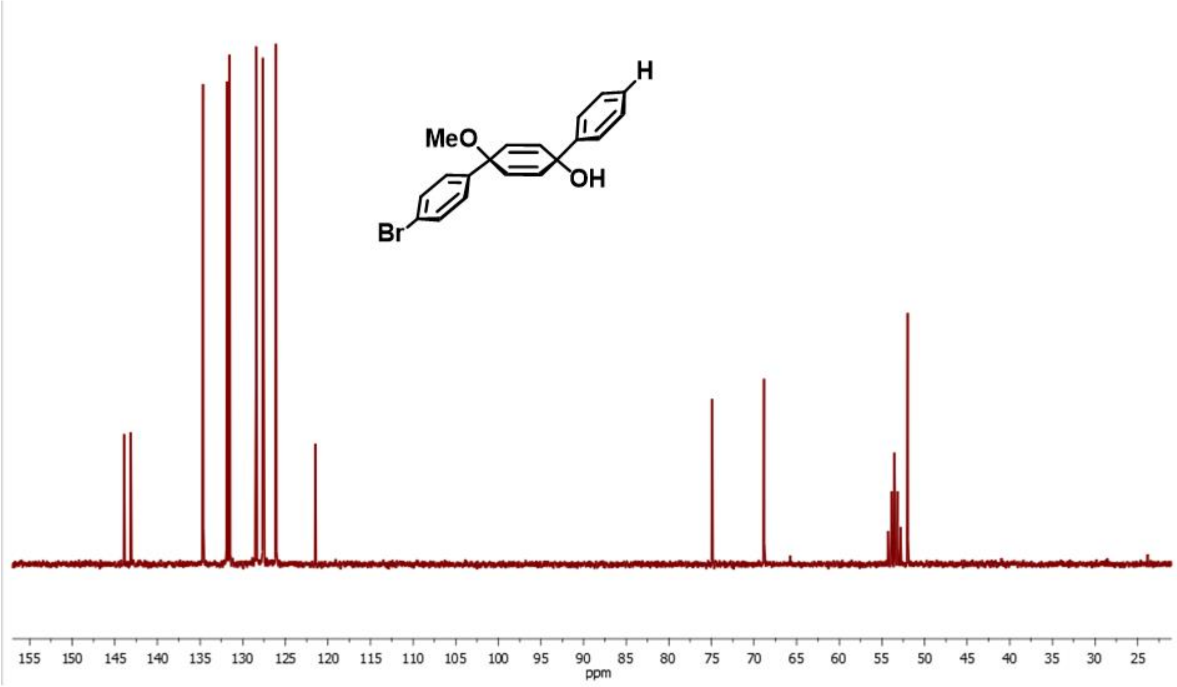

Figure S22: ^{13}C NMR spectrum of ***anti*-2** in CD_2Cl_2 (75 MHz, 298 K).

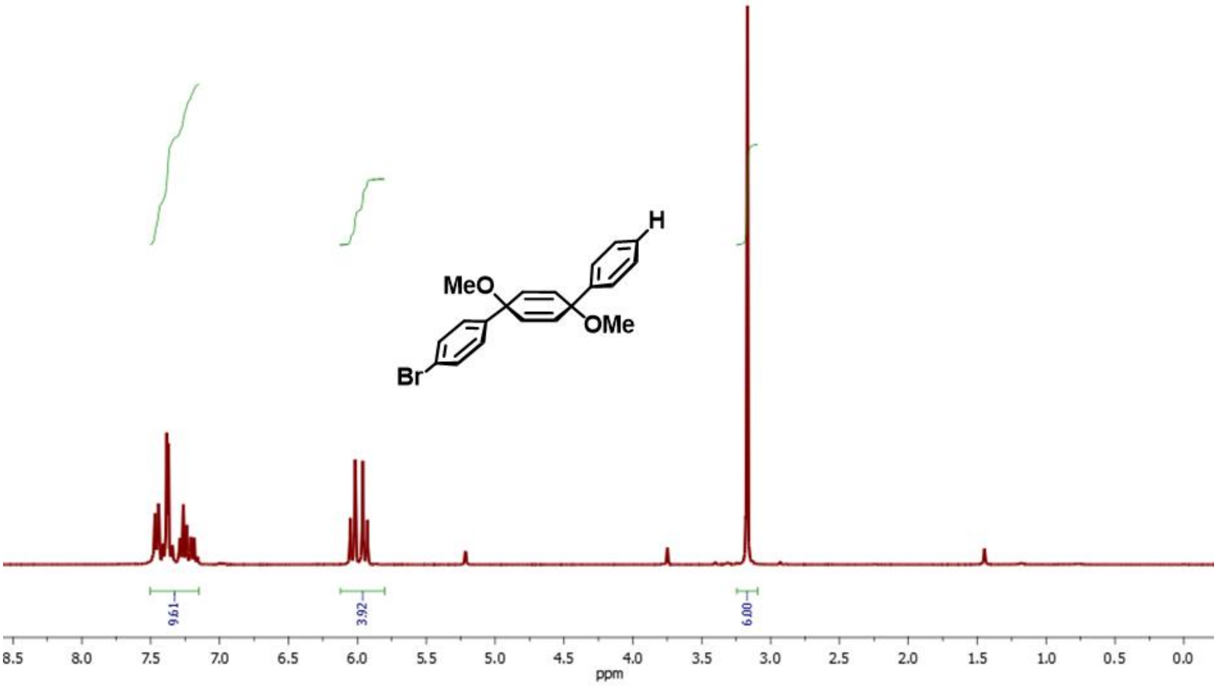

Figure S23: ^{1}H NMR spectrum of ***anti*-3** in CD_2Cl_2 (300 MHz, 298 K).

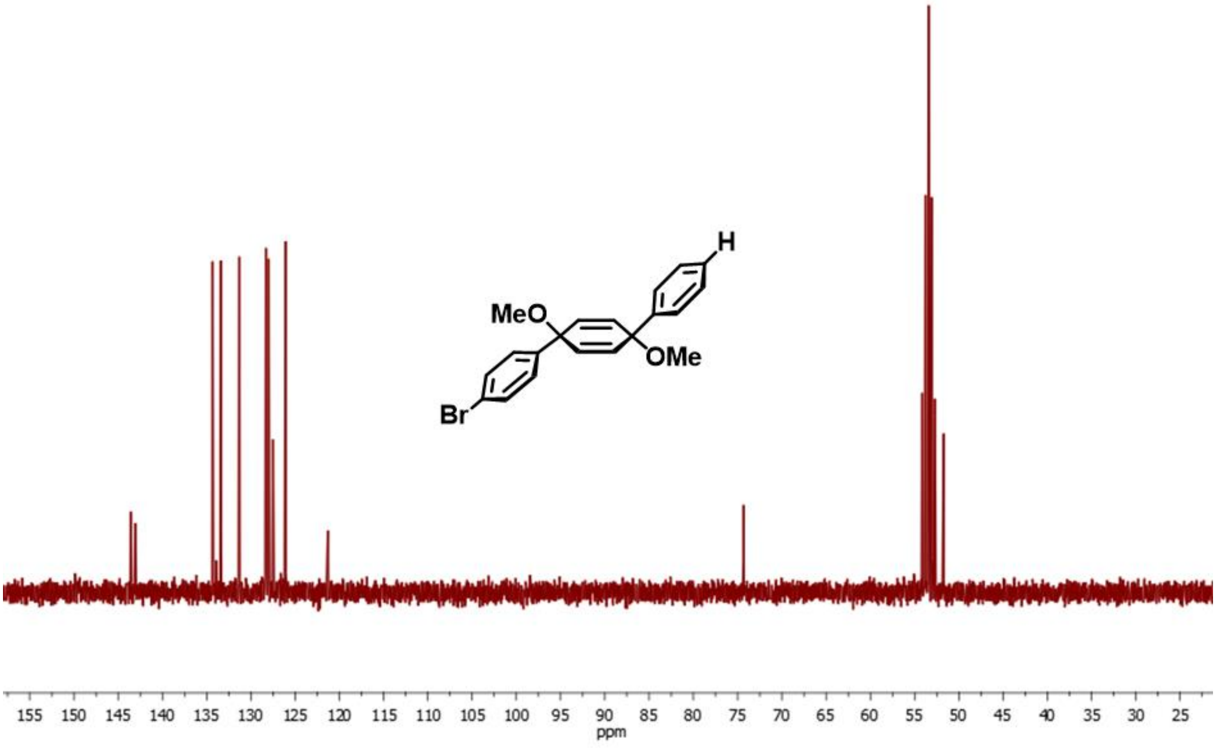

Figure S24: ^{13}C NMR spectrum of ***anti*-3** in CD_2Cl_2 (75 MHz, 298 K).

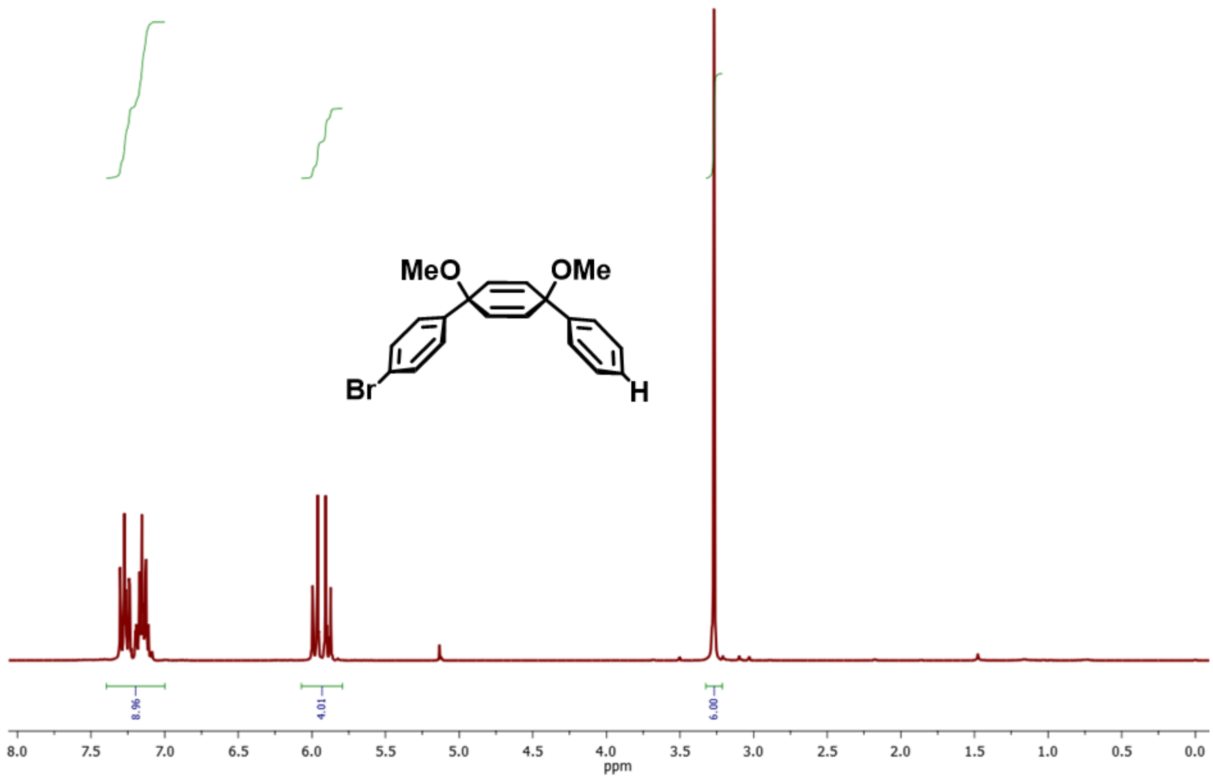

Figure S25: ^{1}H NMR spectrum of ***syn*-3** in CD_2Cl_2 (300 MHz, 298 K).

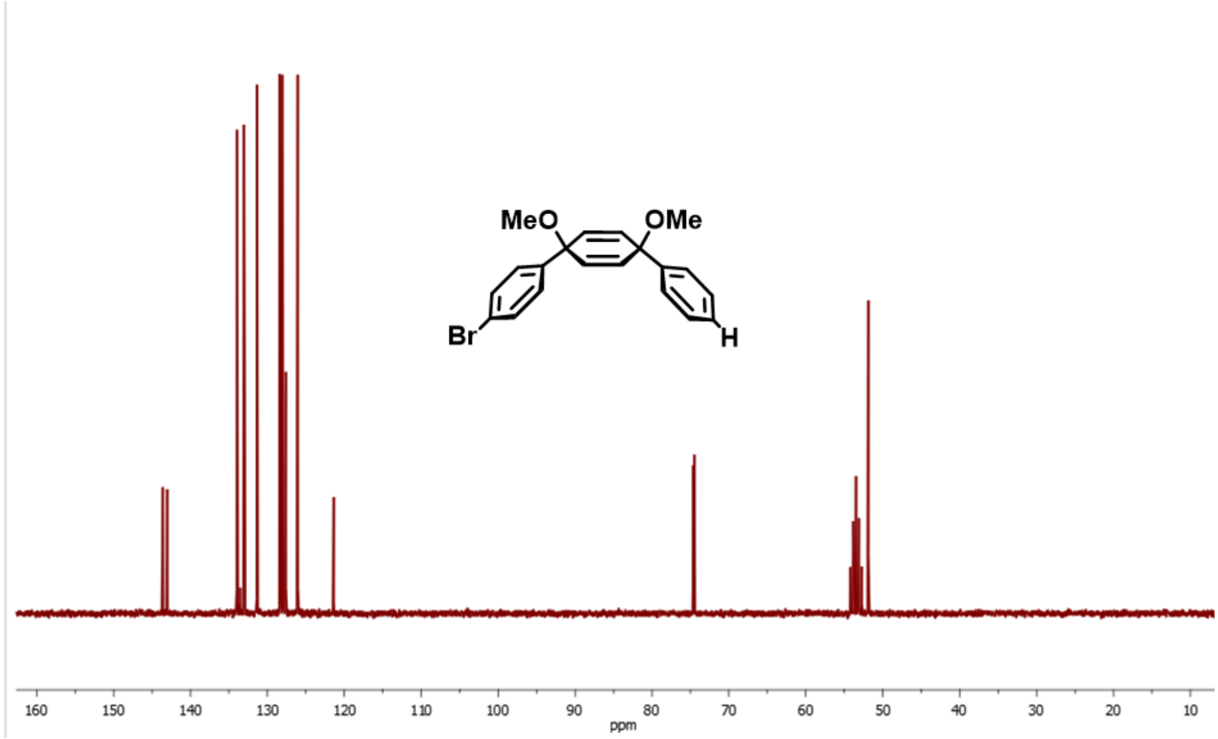

Figure S26: ^{13}C NMR spectrum of ***syn*-3** in CD_2Cl_2 (75 MHz, 298 K).

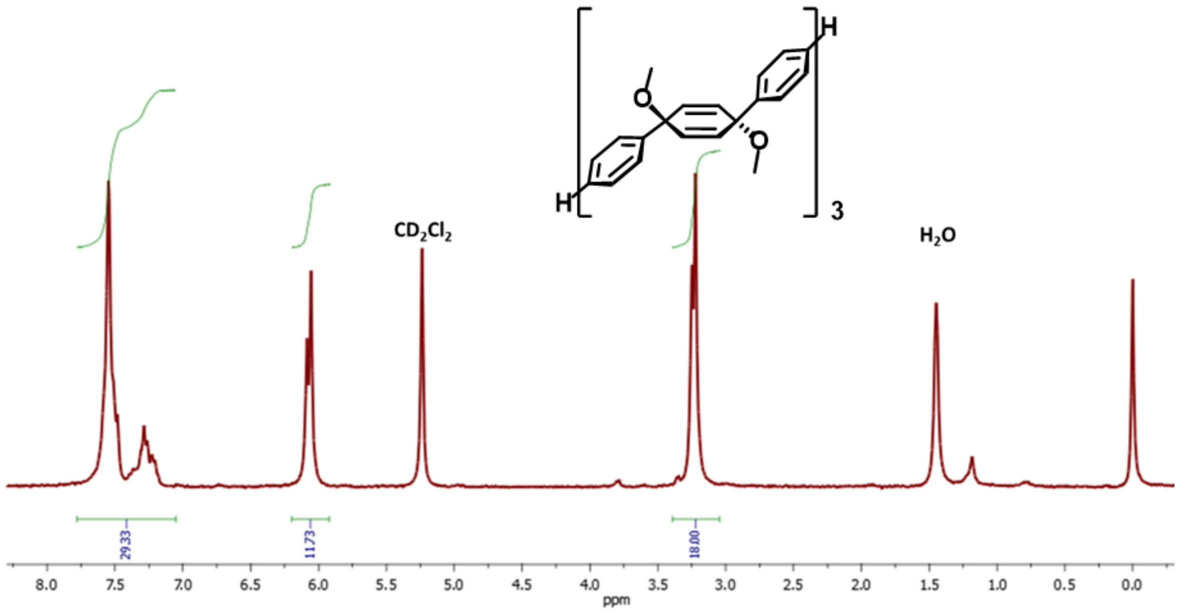

Figure S27: ^{1}H NMR spectrum of **9PP**$_{\textbf{\textit{anti,pre}}}$ in CD_2Cl_2 (300 MHz, 298 K).

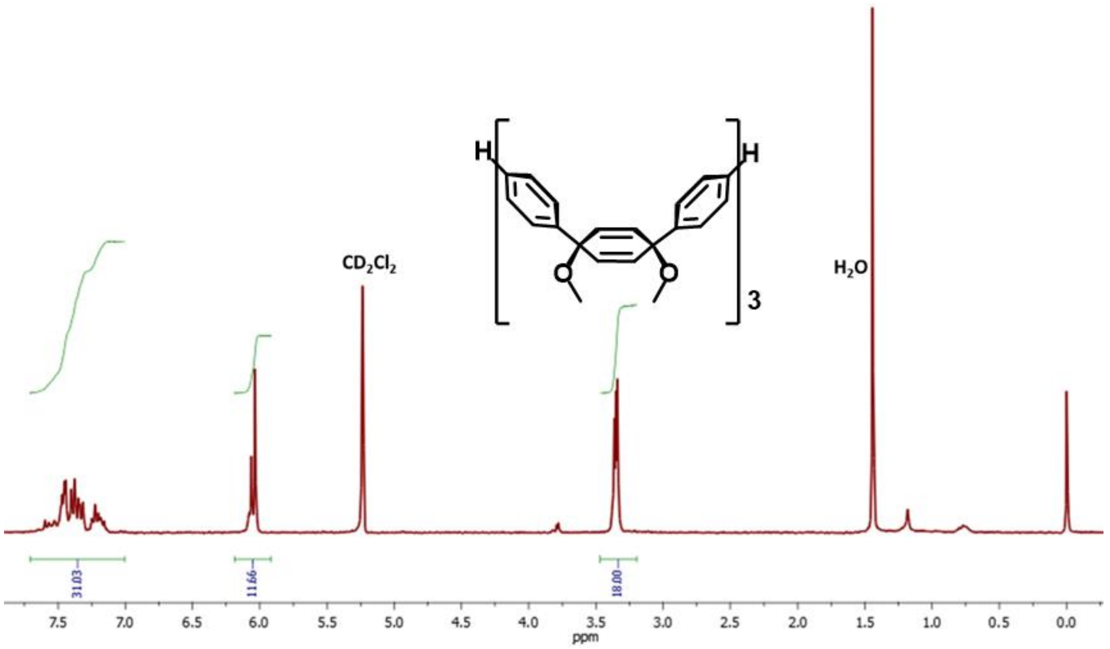

Figure S28: ^{1}H NMR spectrum of **9PP**$_{\textbf{\textit{syn,pre}}}$ in CD_2Cl_2 (300 MHz, 298 K).

7 Solid-State NMR Spectra

Solid-State ^{13}C NMR spectra of **9PP*syn/anti,pre*** and **9PP*syn,anti***, obtained from thermal aromatization in the bulk at 300 °C for 5 h. The ^{13}C spectra of **9PP*syn,anti*** shows good agreement with other reported *para*-phenylenes.[15] Fitting of the ^{13}C signals in the product spectra confirms the chain length for both, **9PP*syn*** and **9PP*anti***, of 9 phenylene units.

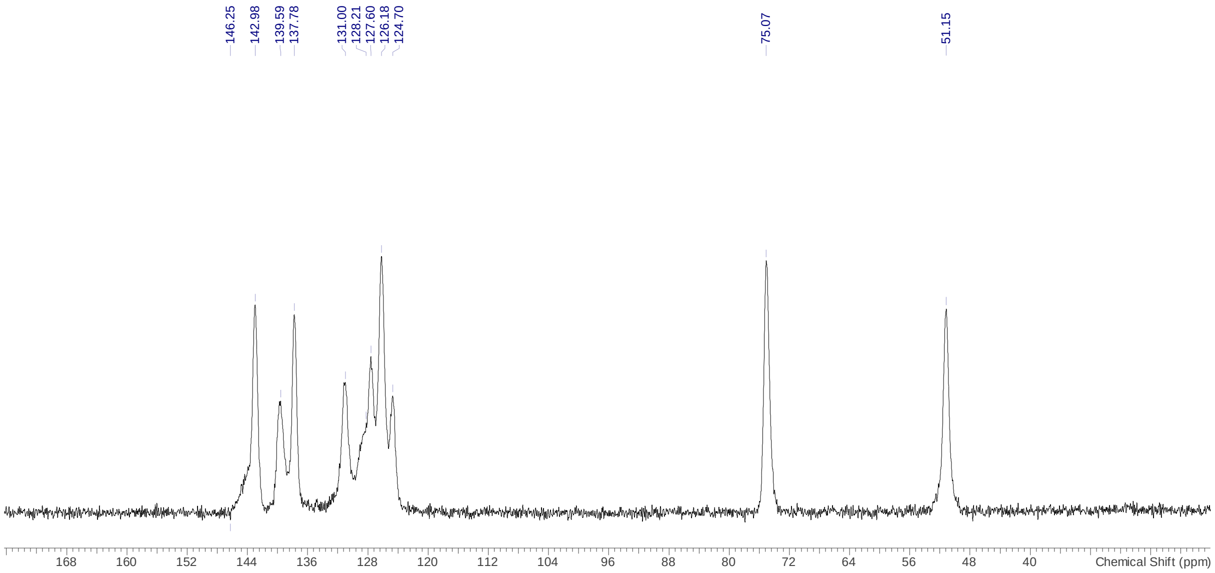

Figure S29: ^{13}C{^{1}H}-CP/MAS-NMR spectra of **9PP*anti,pre*** (25 kHz, 298 K).

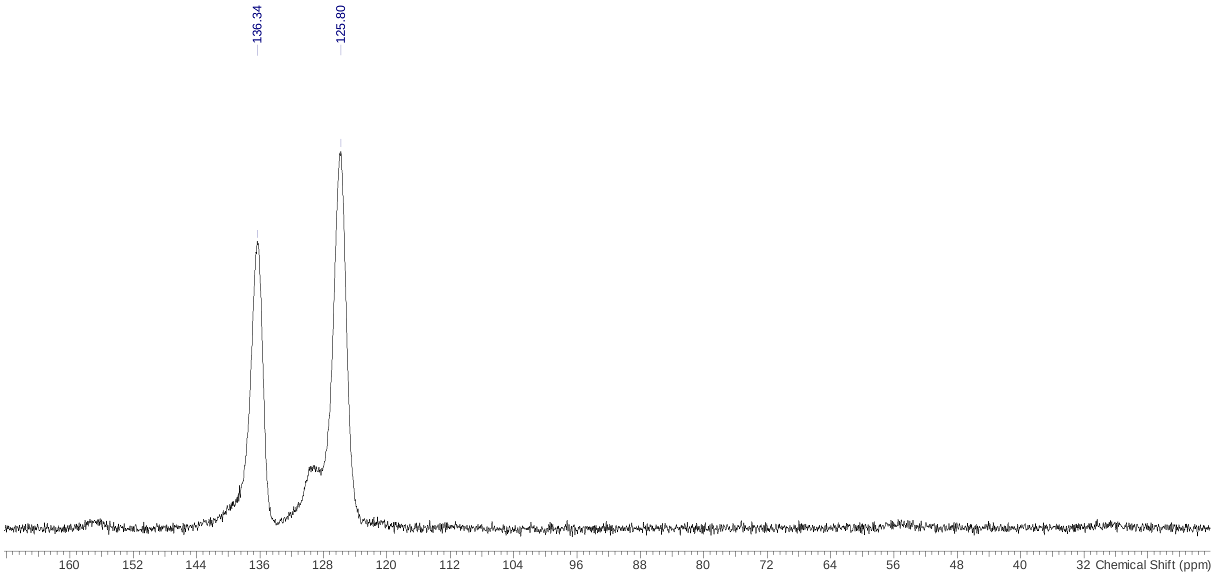

Figure S30: ^{13}C{^{1}H}-CP/MAS-NMR spectra of **9PP*anti*** (25 kHz, 298 K).

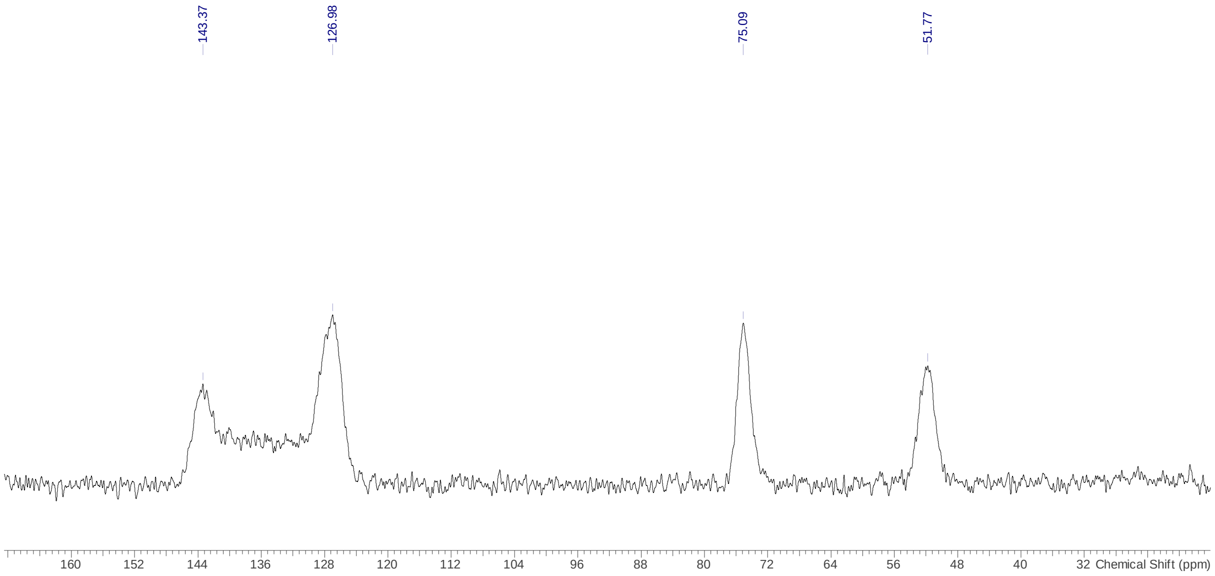

Figure S31: $^{13}C\{^{1}H\}$-CP/MAS-NMR spectra of **9PP*syn,pre*** (25 kHz, 298 K).

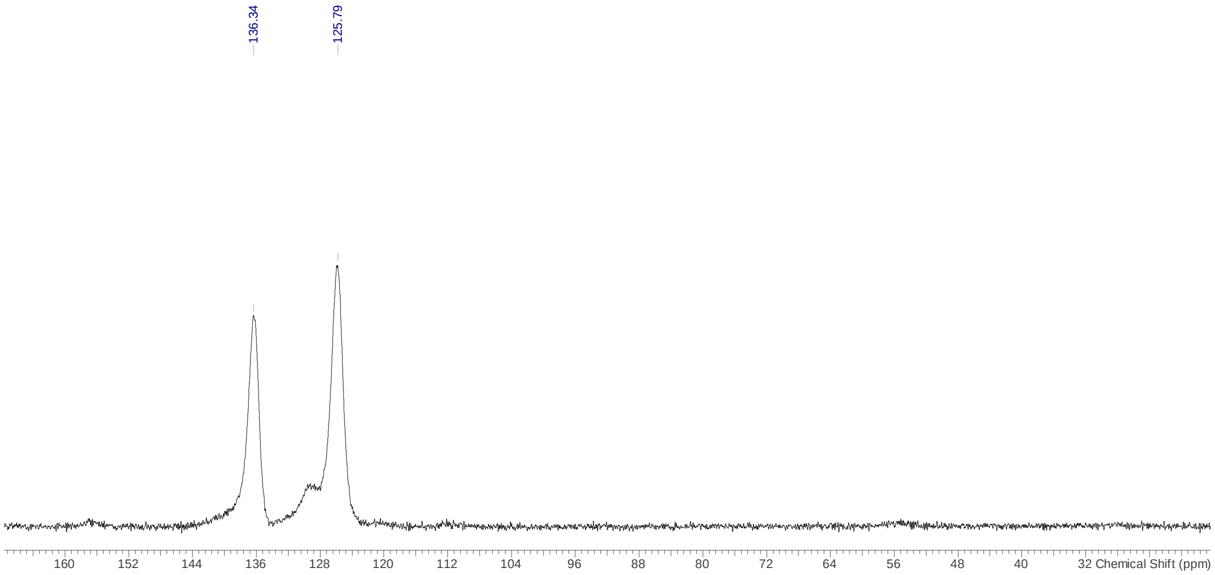

Figure S32: $^{13}C\{^{1}H\}$-CP/MAS-NMR spectra of **9PP*syn*** (25 kHz, 298 K).

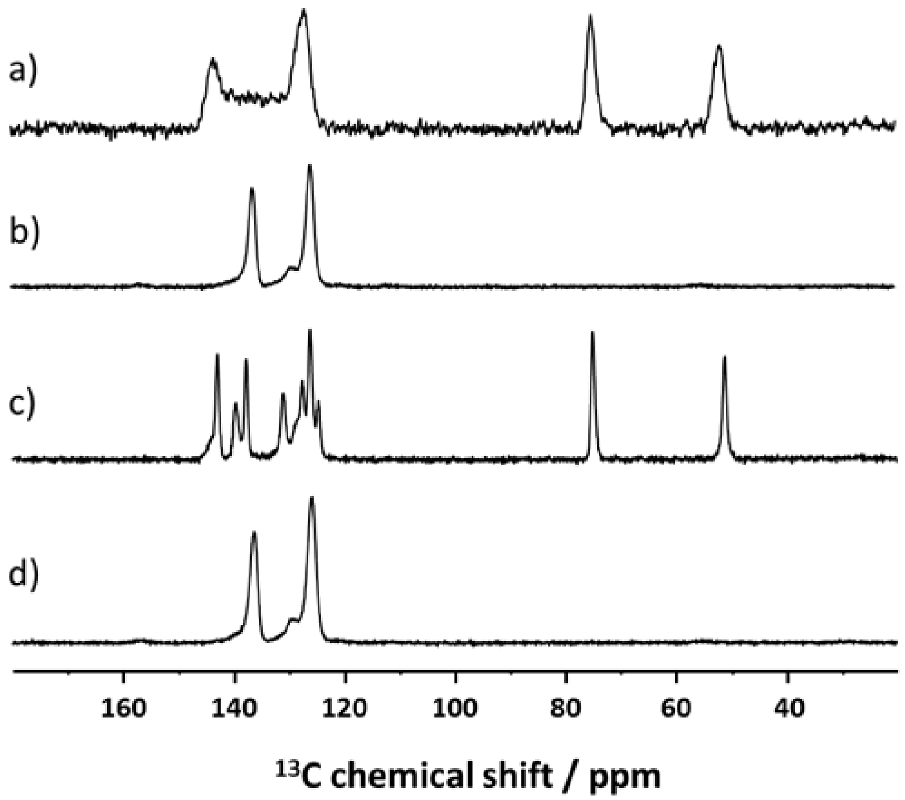

Figure 33. ^{13}C-CP/MAS-NMR spectra of a) **9PP**$_{syn,pre}$, b) **9PP**$_{syn}$, c) **9PP**$_{anti,pre}$ and d) **9PP**$_{anti}$ recorded with 3ms contact time. The ^{13}C-CP/MAS spectra clearly demonstrate the aromatization of the products.

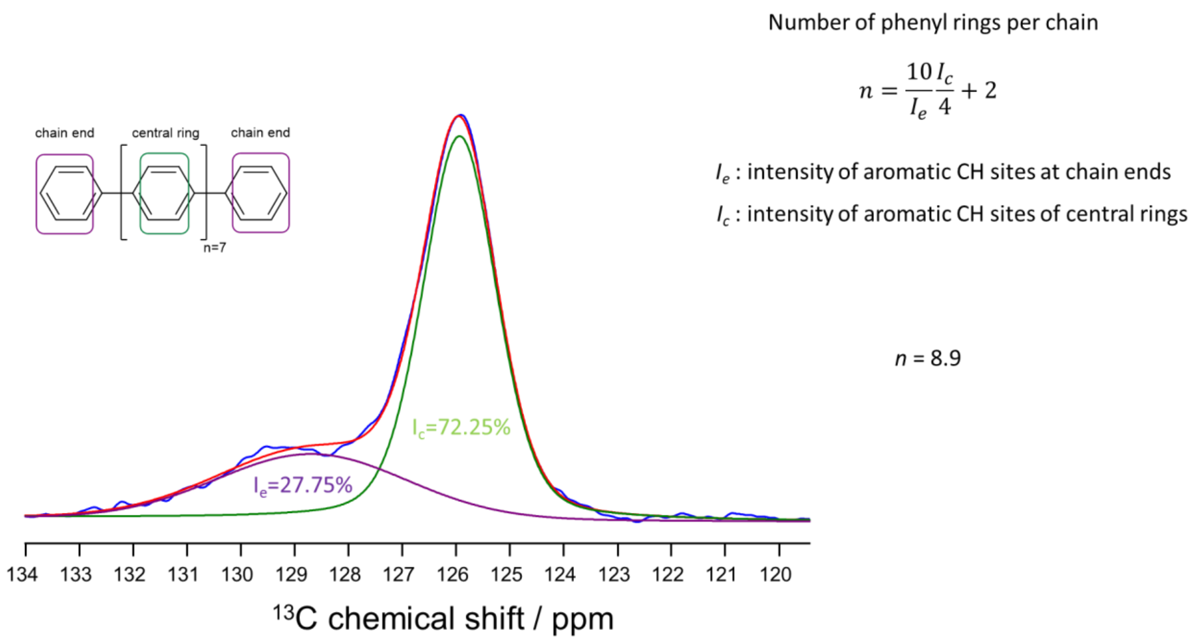

Figure 34. Fit of the ^{13}C-**C**H signal intensity for the chain ends and central rings confirms the chain length of 9 rings for **9PP***syn.*

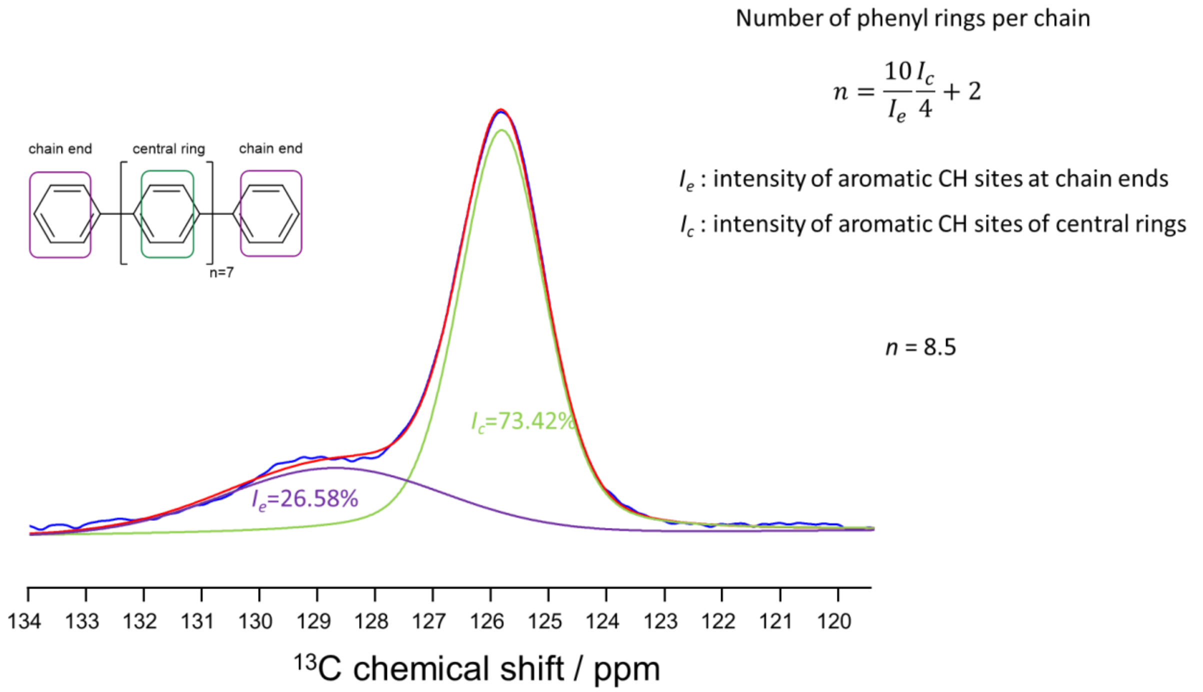

Figure 35. Fit of the ^{13}C-**C**H signal intensity for the chain ends and central rings confirms the chain length of 9 rings for **9PP***anti.*

References:

[1] G. R. Fulmer, A. J. M. Miller, N. H. Sherden, H. E. Gottlieb, A. Nudelman, B. M. Stoltz, J. E. Bercaw, K. I. Goldberg, NMR Chemical Shifts of Trace Impurities: Common Laboratory Solvents, Organics, and Gases in Deuterated Solvents Relevant to the Organometallic Chemist. *Organomet.* **2010**, *29*, 2176–2179.

[2] M. J. Frisch, G. W. Trucks, H. B. Schlegel, G. E. Scuseria, M. A. Robb, J. R. Cheeseman, G. Scalmani, V. Barone, G. A. Petersson, H. L. Nakatsuji, X., M. Caricato, A. V. Marenich, J. Bloino, B. G. Janesko, R. Gomperts, B. Mennucci, H. P. Hratchian, J. V. Ortiz, A. F. Izmaylov, J. L. Sonnenberg, D. Williams-Young, F. L. Ding, F., F. Egidi, J. Goings, B. Peng, A. Petrone, T. Henderson, D. Ranasinghe, V. G. Zakrzewski, J. Gao, N. Rega, G. Zheng, W. Liang, M. Hada, M. Ehara, K. Toyota, R. Fukuda, J. Hasegawa, M. Ishida, T. Nakajima, Y. Honda, O. Kitao, H. Nakai, T. Vreven, K. Throssell, J. A. Montgomery, Jr., J. E. Peralta, F. Ogliaro, M. J. Bearpark, J. J. Heyd, E. N. Brothers, K. N. Kudin, V. N. Staroverov, T. A. Keith, R. Kobayashi, J. Normand, K. Raghavachari, A. P. Rendell, J. C. Burant, S. S. Iyengar, J. Tomasi, M. Cossi, J. M. Millam, M. Klene, C. Adamo, R. Cammi, J. W. Ochterski, R. L. Martin, K. Morokuma, O. Farkas, J. B. Foresman, D. J. Fox, *Gaussian 09W, Version 8.0, Gaussian Inc. Wallingford, USA* **2009**.

[3] A. D. Becke, Density-functional thermochemistry. III. The role of exact exchange *J. Chem. Phys.* **1993**, 98, 7, 5648–5652.

[4] P. J. Stephens, F. J. Devlin, C. F. N. Chabalowski, and M. J. Frisch, Ab initio calculation of vibrational absorption and circular dichroism spectra using density functional force fields. *J. Phys. Chem.* **1994**, 98, 45, 11623-11627.

[5] W. Theiss, Scout 4.56, W.Theiss Hard- and Software: 2018.

[6] K. Jacobs, S. H. Thin Liquid Polymer Films Rupture via Defects. *Langmuir* **1998**, *14*, 965-969.

[7] R. Seemann, S. H., K. Jacobs. Dewetting Patterns and Molecular Forces: A Reconciliation. *Phys. Rev. Lett.* **2001**, *86*, 5534-5537.

[8] Thompson, C. V. Solid-State Dewetting of Thin Films *Annu. Rev. Mater. Res.* **2012**, *42*, 399–434.

[9] G. Reiter, Dewetting of Thin Polymer Films. *Phys. Rev. Lett.* **1992**, *68*, 75-78.

[10] L. Xue , Y. H. Inhibition of dewetting of thin polymer films. *Prog. Mater. Sci.* **2012**, *57*, 947–979.

[11] J. Gierschner, S. Y. Park, *J. Mater. Chem. C* **2013**, 1, 5818–5832.

[12] F.C. Spano, Annu. *Rev. Phys. Chem.* **2006**, 57, 217−243.

[13] A. Kadashchuk, A. Andreev, H. Sitter, N. Sariciftci, Y. Skryshevski, Y. Piryatinski, I. Blonsky, D. Meissner, *Adv. Funct. Mater.* **2004**, 14, 970-978.

[14] A. Piaggi, G. Lanzani, G. Bongiovanni, A. Mura, W. Graupner, F. Meghdadi, G. Leising, M. Nisoli, *Phys. Rev. B* **1997**, 56, 10 133.

[15] K. Erdmann, W. Czerwinski, B.C. Gerstein, M. Pruski, *J. Polym. Sci. B Polym. Phys.* **1994**, 32, 1961-1968.
